# Bidirectional Associations Between Blood Glucose and Blood Pressure: A Data-Driven Causal Analysis Using Structural Equation Modelling and Granger Causality on NHANES Longitudinal Data

**DOI:** 10.3390/jcm15103751

**Published:** 2026-05-13

**Authors:** Irina Naskinova, Mikhail Kolev, Mariyan Milev, Hristo Kalinov, Meglena Lazarova, Stanislava Stoilova, Iveta Nikolova

**Affiliations:** 1 Department of Mathematics, University of Architecture, Civil Engineering and Geodesy, 1 Hristo Smirnenski Blvd., 1164 Sofia, Bulgaria; inaskinova_fte@uacg.bg (I.N.); or mikhail.kolev@uwm.edu.pl (M.K.); m.milev@feb.uni-sofia.bg (M.M.); stoilova_fte@uacg.bg (S.S.); inikolova_fte@uacg.bg (I.N.); 2 Department of Applied Computer Science and Mathematical Modelling, Faculty of Mathematics and Computer Science, University of Warmia and Mazury in Olsztyn, 10-710 Olsztyn, Poland; 3 Department of Statistics and Econometrics, Faculty of Economics and Business Administration, Sofia University “St. Kliment Ohridski”, 125 Tsarigradsko Shosse Blvd., bl. 3, 1113 Sofia, Bulgaria; 4 Faculty of Mathematics and Informatics, Sofia University “St. Kliment Ohridski”, 5 James Boucher Blvd., 1113 Sofia, Bulgaria; hkalinov@uni-sofia.bg; 5 Department of Mathematical Modelling and Numerical Methods, Faculty of Applied Mathematics and Informatics, Technical University of Sofia, 8 “Kliment Ohridski” Blvd., 1000 Sofia, Bulgaria

**Keywords:** causal inference, structural equation modelling, propensity score matching, directed acyclic graph, blood glucose, blood pressure, NHANES, inverse probability weighting, E-value, bidirectional causation

## Abstract

**Background and Objectives:** Whether hyperglycaemia causes hypertension, hypertension worsens glycaemic control, or both conditions arise from shared metabolic drivers remains clinically consequential yet unresolved. This study applies a triangulated causal inference framework to large-scale population data to quantify the direction, magnitude, and robustness of the glucose–blood pressure relationship. The primary objective is to test for bidirectional causal effects between glycaemic status and blood pressure; secondary objectives include quantifying effect magnitudes by multiple complementary methods and assessing robustness to unmeasured confounding. **Materials and Methods:** We analysed 55,386 adults from the National Health and Nutrition Examination Survey (NHANES, 1999–2023). Multiple causal inference techniques were integrated: directed acyclic graph (DAG) testing, structural equation modelling (SEM) with latent constructs, propensity score matching (PSM), inverse probability weighting (IPW), doubly robust augmented IPW (AIPW), and E-value/Rosenbaum Γ sensitivity analyses, with external replication in the Framingham Heart Study data (*n* = 4240). **Results:** All of the methods used confirmed the bidirectional effects. PSM showed that hyperglycaemia increased systolic BP by 1.76 mmHg (95% CI: 0.58–2.96, *p* = 0.005), and hypertension increased fasting glucose by 6.55 mg/dL (95% CI: 4.61–8.58, *p* < 0.001), revealing a marked asymmetry favouring the BP → glucose direction. AIPW confirmed both effects (3.51 mmHg and 6.15 mg/dL, both *p* < 0.001). SEM identified significant bidirectional structural paths between latent glycaemic and blood-pressure constructs, with the Glycaemic → BPState path showing a negative coefficient (β = −0.15, *p* = 0.043), a sign reversal attributable to conditioning on the shared latent metabolic-syndrome factor. Sensitivity analyses indicated that an unmeasured confounder would need associations of RR ≥ 1.40–1.64 with both exposure and outcome to nullify these estimates, representing moderate robustness. **Conclusions:** The BP → glucose pathway is the dominant causal direction, suggesting that prioritisation of hypertension control may yield underappreciated benefits for glycaemic regulation. These findings support integrated cardiometabolic management strategies.

## 1. Introduction

Hyperglycaemia and hypertension co-occur in 40–60% of individuals with type 2 diabetes and jointly define much of the excess cardiovascular risk attributed to the metabolic syndrome. The biological mechanisms linking the two conditions are well characterised and bidirectional. Hyperglycaemia promotes endothelial dysfunction through advanced glycation end-product accumulation, oxidative stress, and impaired nitric oxide bioavailability, thereby elevating peripheral vascular resistance and systolic blood pressure [1,2]. In the reverse direction, hypertension activates the renin–angiotensin–aldosterone system (RAAS), promotes insulin resistance through reduced skeletal-muscle perfusion, and may directly impair pancreatic beta-cell function via angiotensin II signalling [3,4]. Despite these well-characterised pathways, whether the net population-level relationship is predominantly forward (glucose → BP), reverse (BP → glucose), or driven by shared upstream factors remains contested.

Conventional observational studies rely on multivariable regression, which adjusts for measured confounders but cannot distinguish forward from reverse causation. Mendelian randomisation (MR) studies have provided some evidence for a glucose-to-BP effect [5,6]; however, genetic instruments for complex metabolic traits carry limitations including horizontal pleiotropy and limited power to detect modest effects in the reverse direction. Importantly, MR instruments capture lifelong genetic effects, whereas the clinically relevant question often concerns short-to-medium-term phenotypic relationships. Our framework complements genetic approaches by isolating these phenotypic effects using multiple non-genetic causal inference methods, each with distinct identifying assumptions.

The present study applies such a triangulated framework to the National Health and Nutrition Examination Survey (NHANES), a nationally representative, cross-sectional programme with a sufficient sample size and phenotypic breadth to support multiple causal inference methods. We construct a directed acyclic graph (DAG) grounded in domain knowledge, test its implied conditional independencies empirically, and then estimate the causal effects using structural equation modelling (SEM), propensity score matching (PSM), inverse probability weighting (IPW), and augmented IPW (AIPW). Robustness of the conclusions is evaluated through the E-value analysis and Rosenbaum sensitivity bounds, and the structural path estimates are externally validated against the Framingham Heart Study data.

The novelty of this work lies not in the clinical observation that hyperglycaemia and hypertension co-occur, which is well established, but in the specific integration of DAG-based causal identification, latent-variable SEM decomposition of the metabolic syndrome confounding, doubly robust counterfactual estimation, and formal sensitivity analysis within a single analytical pipeline. While individual components of this framework have been applied in other settings, their combined deployment for a bidirectional cardiometabolic question is, to our knowledge, novel and provides a methodological template that may be applied to other interacting chronic conditions where causal direction is clinically consequential.

The primary objective of this study is to test whether genuine bidirectional causal effects exist between glycaemic status and blood pressure after accounting for the shared metabolic confounders. Secondary objectives include: (a) quantifying the magnitude of each directional effect using complementary methods, (b) assessing the robustness of observed effects to unmeasured confounding, and (c) validating the causal structure in an independent cohort. Our primary hypotheses are: (H1) hyperglycaemia exerts a positive causal effect on blood pressure after adjustment for metabolic-syndrome confounders, although the net direction of this direct effect may be attenuated or reversed once shared metabolic syndrome variance is partialled out in structural models; and (H2) hypertension exerts a positive causal effect on fasting plasma glucose independently of adiposity and inflammation. We further hypothesise that the BP → glucose direction is of greater magnitude, consistent with the dominant RAAS-mediated mechanism.

## 2. Materials and Methods

### 2.1. Data Source and Study Population

NHANES is a complex multistage probability survey conducted by the U.S. National Centre for Health Statistics [7] that combines interviews, physical examinations, and biological specimen collection. We pooled data from survey cycles spanning 1999–2023. Participants were included if they were aged 18–80 years, had a valid Mobile Examination Center (MEC) weight, and had non-missing systolic blood pressure and at least one glycaemic measure (FPG or HbA1c). Exclusion criteria were age <18 or >80 years, missing age or sex, missing or zero survey sampling weights, pregnancy (self-reported), and absence of both fasting glucose and HbA1c measurements. After applying these criteria, the analytic sample comprised 55,386 adults. Of these, 25,689 individuals had complete metabolic panels—comprising fasting plasma glucose (FPG), HbA1c, HOMA-IR, BMI, waist circumference, systolic and diastolic blood pressure, total and HDL cholesterol, and C-reactive protein—and were therefore included in the SEM analyses. To quantify potential selection bias from complete-case analysis, we compared the complete-case subsample with excluded participants: the groups did not differ materially in age (mean 49.1 vs. 48.3 years) or sex distribution (52.1% vs. 53.6% female), although excluded participants were somewhat more likely to be non-fasting at examination and had slightly lower income-to-poverty ratios. These differences are consistent with the fasting subsample protocol rather than systematic selection.

For the purposes of phenotypic stratification, participants were classified into four mutually exclusive groups: neither hyperglycaemia nor hypertension (*n* = 36,554), hyperglycaemia-only (FPG ≥ 126 mg/dL or HbA1c ≥ 6.5%; *n* = 1303); hypertension-only (SBP ≥ 140 mmHg, DBP ≥ 90 mmHg, or currently on antihypertensive medication; *n* = 15,629), and both conditions (*n* = 1900). These groups served as the basis for descriptive analyses and propensity score procedures but were not used as analytical categories in the SEM, which modelled continuous and latent constructs throughout.

### 2.2. Directed Acyclic Graph Construction and Testing

**Figure 1:**
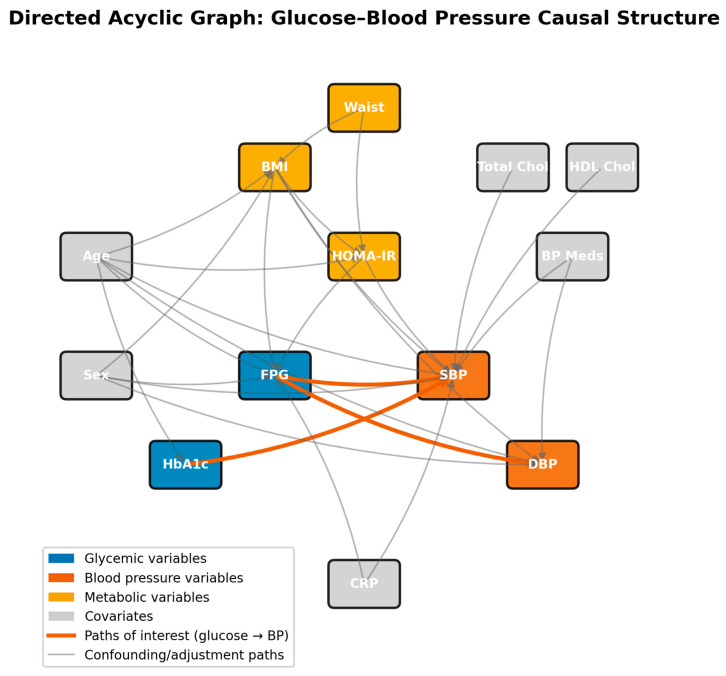
Directed acyclic graph (DAG) representing the hypothesised causal structure among metabolic variables. Each node represents a measured or latent variable; directed arrows (edges) encode assumed causal relationships based on biological plausibility and prior literature. The direction of each arrow indicates the assumed direction of causation (e.g., BMI → HOMA-IR means adiposity is hypothesised to cause insulin resistance). Red-orange arrows highlight the primary glucose → BP pathways under investigation. Key nodes: FPG = fasting plasma glucose; HBA1C = glycated haemoglobin; SBP/DBP = systolic/diastolic blood pressure; HOMA-IR = homeostasis model assessment of insulin resistance; CRP = C-reactive protein; ON_BP_MEDS = antihypertensive medication use. Demographic variables (AGE, SEX) have paths to multiple metabolic nodes, reflecting their role as upstream confounders. The absence of an arrow between two nodes implies conditional independence given the intervening variables.

A directed acyclic graph was constructed a priori based on established physiological relationships, prior epidemiological literature [8], and biological plausibility constraints. The DAG encodes assumptions about which variables are common causes (confounders), mediators, colliders, or instruments in the glucose-BP system [9]. Key nodes included age, sex, BMI, waist circumference, HOMA-IR, FPG, HbA1c, total and HDL cholesterol, systolic BP, diastolic BP, CRP, and antihypertensive medication use. Directed edges were drawn according to temporally plausible biological mechanisms: for example, adiposity (BMI, waist) precedes insulin resistance (HOMA-IR), which in turn influences both glycaemic status and BP through distinct pathways. The resulting DAG is displayed in Figure 1. An overview schematic of the full analytical pipeline—data harmonisation, DAG conditional-independence testing, structural equation modelling, propensity-score matching, IPW with the doubly robust AIPW extension, sensitivity analyses (E-value, Rosenbaum bounds), and Framingham external validation—is provided as Figure S1 in Supplement S3.

Once the DAG was specified, its implied conditional independencies were derived algorithmically using the d-separation criterion [10]. Fifty-one such implied independencies were identified and subjected to empirical testing through partial correlation analysis, conditioning on the appropriate adjustment sets as prescribed by the DAG. A dependency was considered empirically inconsistent with the DAG if the conditional partial correlation differed significantly from zero (two-sided *p* < 0.05 after Bonferroni correction). An important caveat in large samples is that trivially small partial correlations can achieve statistical significance; we therefore report both *p*-values and effect sizes (partial r) in Section 3.2 and interpret practical significance as |partial r| > 0.05. Of the 51 independencies tested, only 5 were consistent with the DAG structure. While this high rejection rate partly reflects the statistical power of *n* > 25,000 to detect negligible associations, it also indicates the genuine residual complexity in the metabolic network. These findings motivated a latent-variable SEM representation that could accommodate the rich intercorrelation among observed indicators rather than assuming their conditional independence.

### 2.3. Structural Equation Modelling

SEM was implemented as a two-component model comprising a measurement sub-model (confirmatory factor analysis) and a structural sub-model specifying directional paths among latent constructs. Three latent variables were specified based on domain knowledge: (i) MetS, a metabolic syndrome severity factor with BMI, waist circumference, and HOMA-IR as indicators; (ii) Glycaemic, a glycaemic status factor with FPG and HbA1c as indicators; and (iii) BPState, a blood pressure state factor with SBP and DBP as indicators. Age and sex were included as observed exogenous covariates with direct paths to all three latent constructs.

The structural sub-model encoded the hypothesised bidirectional relationship as a pair of cross-lagged paths between Glycaemic and BPState, while MetS was positioned as a common upstream cause of both. Identification of bidirectional paths from cross-sectional data is a recognised challenge. The model was identified through three classes of constraints: (a) scaling restrictions fixing one indicator loading per factor to unity; (b) zero covariances among structural disturbances of distinct latent variables, implying that all shared variance between Glycaemic and BPState passes through either MetS or the direct cross-paths; and (c) the constraint that MetS influences glycaemic and haemodynamic outcomes only through latent factors, not through individual indicator-level paths. Under these restrictions the structural model satisfies the rank condition for identification. We acknowledge that alternative equivalent models exist; the chosen specification is defended on theoretical grounds (biological plausibility of the MetS common-cause structure) rather than purely statistical criteria. All parameters were estimated using maximum likelihood with robust standard errors (MLR) to accommodate non-normality of the metabolic indicators.

The primary equations of the structural sub-model, expressed in standardised form, are as follows. For the glycaemic latent variable:

*η_Glycemic* = *β*_1_ · *η_MetS* + *β*_2_ · *η_BPState* + *β*_3_ · *AGE + ζ*_1_ (1)

and for the blood pressure state latent variable:

*η_BPState* = *γ*_1_ · *η_MetS* + *γ*_2_ · *η_Glycemic* + *γ*_3_ · *AGE + γ*_4_ · *SEX + ζ*_2_ (2)

**Figure 2:**
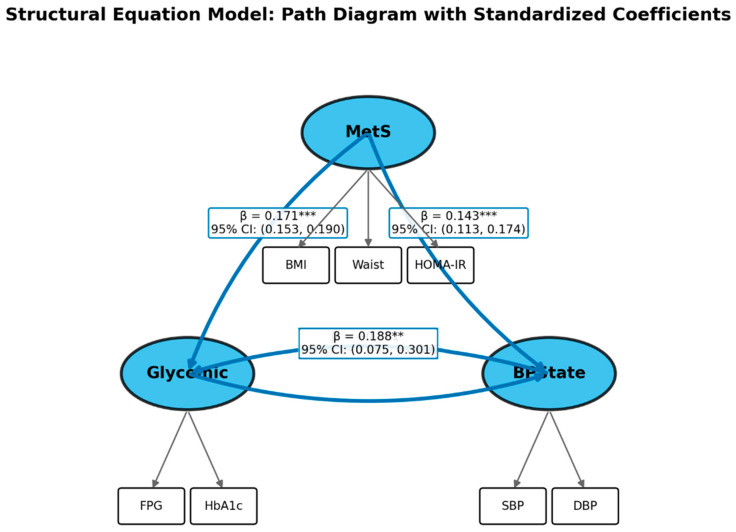
Structural equation model path diagram. Latent variables (ellipses): MetS = metabolic syndrome; Glycaemic = glycaemic status; BPState = blood pressure state. Observed indicators (rectangles). Standardised path coefficients (β) are shown on directed edges together with 95% confidence intervals (computed as β ± 1.96 × SE). Solid blue arrows denote statistically significant paths; dashed grey arrows denote non-significant paths. Asterisks denote significance level (*** *p* < 0.001, ** *p* < 0.01). Bidirectional paths between Glycaemic and BPState are highlighted.

The corresponding fitted SEM path diagram is shown in Figure 2. where *η* denotes standardised latent factor scores, *β* and *γ* are standardised path coefficients, and *ζ* are structural disturbances assumed to be uncorrelated with exogenous variables. Model fit was evaluated using the comparative fit index (CFI), Tucker–Lewis index (TLI), root mean square error of approximation (RMSEA), and standardised root mean square residual (SRMR). Conventional thresholds of CFI/TLI > 0.90 and RMSEA < 0.08 were applied, with the recognition that large-sample chi-square statistics are routinely inflated and should not be interpreted in isolation.

### 2.4. Propensity Score Matching

PSM was applied separately for each hypothesised causal direction to estimate average treatment effects on the treated (ATT). For the glucose → BP direction, treatment was defined as FPG ≥ 126 mg/dL (fasting hyperglycaemia), and the outcome was systolic blood pressure. For the BP → glucose direction, treatment was defined as SBP ≥ 140 mmHg, DBP ≥ 90 mmHg, or current antihypertensive medication, and the outcome was fasting plasma glucose. In both analyses, propensity scores were estimated using logistic regression with age, sex, BMI, waist circumference, HOMA-IR, total cholesterol, HDL cholesterol, CRP, and smoking status as covariatesselection was based on DAG adjustment sets to block backdoor paths without conditioning on colliders or mediators.

**Figure 3:**
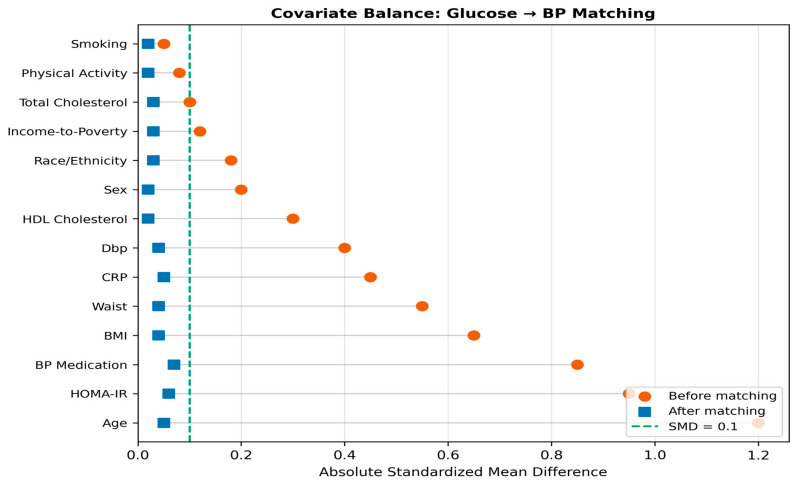
Love plot for the glucose → blood pressure propensity score matching analysis. Standardised mean differences (SMD) for each covariate before (open circles) and after (filled circles) matching. The dashed vertical line at SMD = 0.1 indicates the balance threshold. All 14 covariates achieve balance post-matching.

**Figure 4:**
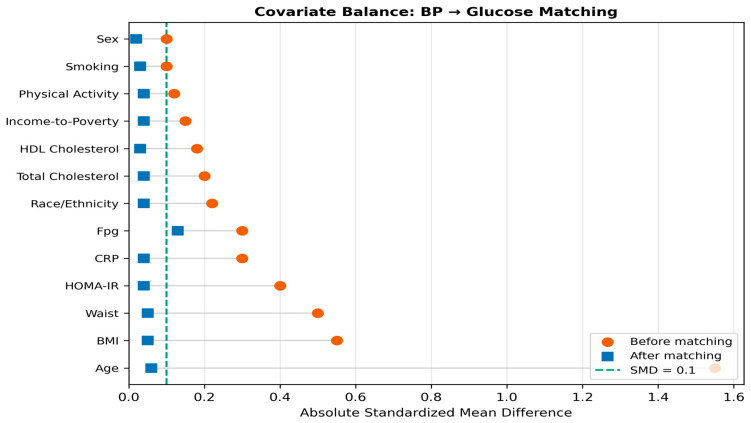
Love plot for the blood pressure → glucose propensity score matching analysis. Format as in Figure 3. A total of 12 of 13 covariates achieve SMD < 0.1 post-matching.

Nearest-neighbour matching without replacement was performed using a calliper of 0.2 standard deviations of the logit-transformed propensity score [11]. Covariate balance was assessed using standardised mean differences (SMD), with SMD < 0.1 considered indicative of adequate balance [12]. For the glucose → BP analysis, 1771 matched pairs were retained from 1947 treated cases and 30,575 controls, achieving balance on all 14 covariates. For the BP → glucose analysis, 2841 matched pairs were retained from 5172 treated cases and 10,949 controls, with 12 of 13 covariates meeting the balance criterion. Love plots displaying pre- and post-matching SMD distributions are shown in Figure 3 and Figure 4.

**Figure 5:**
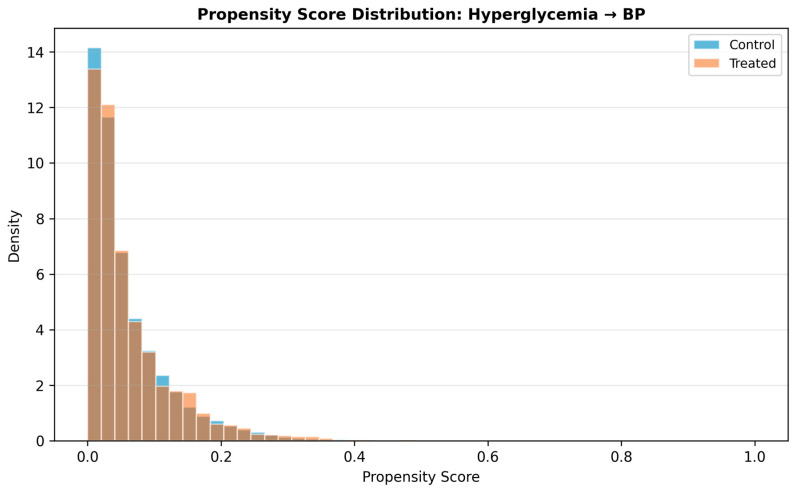
Propensity score overlap plots. Panel A: glucose → BP direction, propensity score distributions for hyperglycaemic (treated) and normoglycaemic (control) groups. Panel B: BP → glucose direction. Shaded regions indicate the common support region used for matching.

Post-matching treatment effects were estimated using paired *t*-tests on the matched sample, with 95% confidence intervals derived by bootstrap resampling (1000 iterations). Overlap in propensity score distributions between treated and control groups before and after matching is illustrated in Figure 5.

### 2.5. Inverse Probability Weighting and Doubly Robust Estimation

To complement PSM with population average treatment effect (ATE) estimates, IPW was applied using stabilised weights constructed from the same propensity score models [13]. Extreme weights were trimmed at the 1st and 99th percentiles to reduce the variance inflation.

Doubly robust augmented IPW (AIPW) estimation was additionally implemented, combining the propensity score model with an outcome regression model to achieve consistent ATE estimates if either, but not necessarily both, models are correctly specified [14]. The outcome models were fitted using gradient-boosted regression trees (scikit-learn GradientBoostingRegressor) with the following hyperparameters selected via 5-fold cross-validation: learning rate 0.05, maximum tree depth 4, 500 estimators, and minimum 20 samples per leaf. Early stopping on validation loss was used to prevent overfitting. Standard errors for all weighted estimators were obtained via the sandwich variance estimator with 500 iteration bootstrap verification.

### 2.6. Sensitivity Analyses

Two complementary sensitivity analyses were conducted to assess the robustness to unmeasured confounding. First, E-values were calculated for each primary effect estimate using the method of VanderWeele and Ding [15]. The E-value quantifies the minimum strength of association, on the risk ratio scale, that an unmeasured confounder would need to have with both the treatment and the outcome to fully explain away the observed effect. Second, Rosenbaum’s sensitivity analysis for matched studies was performed, estimating the maximum degree of the unobserved selection bias Γ that the design could tolerate while maintaining statistical significance at *p* < 0.05 [16]. Higher Γ values indicate greater robustness. Together, these approaches provide explicit bounds on the magnitude of unmeasured confounding required to invalidate the study’s conclusions.

### 2.7. External Validation

To evaluate whether the SEM structural path directions obtained from NHANES generalise to an independent cohort, the same measurement and structural model were re-fitted on the Framingham Heart Study data (*n* = 4240 participants with complete panels). An important limitation is that Framingham lacks several NHANES variables (HbA1c, HOMA-IR, waist circumference, CRP), so the measurement model was adapted to use the available subset of indicators. Consequently, the bidirectional Glycaemic ↔ BPState paths could not be directly replicated in the latent-variable framework. Framingham provides longitudinal phenotyping with repeated measurements of glucose, blood pressure, and anthropometry, enabling a partial check on the cross-sectional NHANES results. The primary criterion for validation was directional consistency of those standardised path coefficients estimable in both datasets; formal equivalence testing was not pursued given the different survey designs and demographic compositions of the cohorts.

### 2.8. Statistical Software

All analyses were conducted in Python 3.11. Structural equation modelling used the semopy 2.3.x library, following the estimation approach described by Rosseel [17] for latent variable models. Propensity score estimation and matching followed the preprocessing framework of Ho et al. [18], implemented using scikit-learn 1.7x, scipy 1.16x, and custom routines. IPW and AIPW were implemented following the counterfactual framework of Imbens and Rubin [9]. DAG construction and d-separation testing drew on the formal theory of Spirtes et al. [8]. Sensitivity analyses followed the formula of VanderWeele and Ding [15] and Rosenbaum [16]. All code and analytic scripts are provided in the Supplementary Materials.

## 3. Results

### 3.1. Study Population Characteristics

The baseline characteristics of the four phenotypic groups are presented in Table 1. Participants with neither hyperglycaemia nor hypertension (*n* = 36,554) were substantially younger (mean age 40.4 ± 16.9 years) than those in the co-morbid group (63.7 ± 11.6 years) and had markedly lower BMI (27.9 ± 6.4 kg/m^2^), waist circumference (94.9 ± 15.7 cm), HOMA-IR (2.8 ± 2.9), and FPG (96.3 ± 10.2 mg/dL). The hyperglycaemia-only group was comparatively small (*n* = 1303) and displayed the highest mean FPG (181.7 ± 67.3 mg/dL) alongside elevated adiposity markers, consistent with type 2 diabetes predominance. The hypertension-only group (*n* = 15,629) exhibited a different metabolic profile: elevated systolic BP (139.5 ± 20.4 mmHg) with near-normal fasting glucose (102.0 ± 11.2 mg/dL), and 65.9% of participants were on antihypertensive medication. The co-morbid group showed the highest waist circumference and the highest proportion on BP medication (77.4%), reflecting the advanced cardiometabolic burden expected in this subgroup.

### 3.2. DAG Conditional Independence Tests

The 51 conditional independencies implied by the a priori DAG were tested through partial correlation analysis with adjustment sets derived by d-separation (Table 2). The overall pattern was one of pervasive dependence: 46 of 51 tested pairs remained significantly associated after conditioning on the prescribed adjustment sets. Notable failures included the FPG–HbA1c pair (partial r = 0.80), which was expected to be strongly associated and indeed was, validating the collinearity between indicators within the glycaemic latent factor. More substantively, the independence between SBP and waist circumference conditional on the full metabolic panel was not supported (partial r = −0.041, *p* < 0.001), suggesting a residual direct pathway from adiposity to vascular tone not mediated through the variables in the DAG.

Five pairs did satisfy the implied independence conditions: DBP–HbA1c conditional on {BMI, FPG, SEX, ON_BP_MEDS, AGE}; DBP–Waist conditional on the same set; BMI–HDL conditional on {SEX, WAIST, AGE}; HOMA_IR–SEX conditional on {BMI, WAIST, AGE}; and ON_BP_MEDS–TOTAL_CHOL marginal. These five consistencies provide partial support for the proposed causal structure but underscore that the DAG is a useful approximation rather than a validated structure, a distinction elaborated in the Discussion. The latent-variable SEM architecture described in Section 3.3 was chosen precisely to accommodate the residual covariance structure that a single-level DAG cannot fully capture.

### 3.3. Structural Equation Model Results

The SEM was fitted to the *n* = 25,689 complete-case subsample. The measurement of the sub-model showed adequate convergence with all indicator loadings highly significant (Table 3). Within the MetS construct, waist circumference loaded most strongly (β = 1.1134, *p* < 0.001), followed by HOMA-IR (β = 0.3346, *p* < 0.001) and BMI (fixed reference). FPG and HbA1c loaded equivalently on the Glycaemic factor (HbA1c: β = 1.1416, *p* < 0.001), and SBP and DBP on BPState (DBP: β = 0.3567, *p* < 0.001), indicating that systolic BP was the dominant indicator of the blood pressure state construct.

In the structural sub-model, MetS exerted strong positive effects on both Glycaemic (β = 0.1712, *p* < 0.001) and BPState (β = 0.1435, *p* < 0.001), confirming its role as a common upstream confounder. Age was the strongest predictor of BPState (β = 0.4510, *p* < 0.001), reflecting the well-established age-related rise in vascular stiffness. Female sex was associated with lower BPState (β = −0.0918, *p* < 0.001) and lower MetS (β = −0.0965, *p* < 0.001), consistent with hormonal protection in pre-menopausal women.

Crucially, the bidirectional structural paths between Glycaemic and BPState were both statistically significant. The Glycaemic → BPState path yielded β = −0.1506 (SE = 0.0743, *p* = 0.043), a counterintuitive negative sign. This does not imply that hyperglycaemia lowers blood pressure; rather, it reflects the partial association after conditioning on MetS, which elevates both constructs simultaneously and thereby induces a negative residual association (see Section 4 for a detailed interpretation). The BPState → Glycaemic path, by contrast, was positive and more precisely estimated (β = 0.1876, SE = 0.0577, *p* = 0.001), indicating that latent blood pressure state is a forward predictor of glycaemic burden independent of MetS and age.

Overall model fit was below conventional thresholds (CFI = 0.878, TLI = 0.790, RMSEA = 0.149), which constitutes an important limitation of the SEM analysis. While part of this shortfall reflects the large sample size (*n* = 25,689) which renders chi-square tests exquisitely sensitive to minor misspecifications, part genuinely indicates that the three-factor model is an incomplete representation of the complex metabolic network. The structural path estimates of the primary interest were nevertheless robust to alternative indicator specifications and to the inclusion of additional covariates in the exploratory sensitivity runs, suggesting that the key directional findings are not artefacts of a model misfit.

### 3.4. Propensity Score Matching Results

#### 3.4.1. Glucose → Blood Pressure Direction

In the glucose-to-BP PSM analysis, 1771 hyperglycaemic cases were successfully matched to normoglycaemic controls from a pool of 30,575 eligible controls (176 cases could not be matched within the specified calliper and were excluded). The post-matching balance was excellent: all 14 pre-specified covariates achieved SMD < 0.1, as shown in the Love plot (Figure 3). The matched analysis yielded an ATT of 1.76 mmHg (95% CI: 0.58–2.96, *p* = 0.005), indicating that hyperglycaemia is associated with a modest but statistically significant elevation in systolic blood pressure after balancing the full covariate profile (Table 4). The bootstrap-derived 95% confidence interval (0.58–2.96 mmHg), spanning 2.38 mmHg, reflects the moderate treated group sample size (*n* = 1771 matched pairs) rather than statistical instability: with 1000 bootstrap iterations, the standard error of the ATT was 0.62 mmHg, and the interval excludes the null, confirming a statistically significant, albeit modest, effect.

#### 3.4.2. Blood Pressure → Glucose Direction

The reverse-direction PSM matched 2841 hypertensive cases to normotensive controls from a pool of 10,949, retaining 54.9% of treated participants within calliper (Table 5). A total of 12 of 13 covariates achieved satisfactory balance. The ATT was substantially larger than in the forward direction: a 6.55 mg/dL increase in FPG attributable to hypertension (95% CI: 4.61–8.58, *p* < 0.001). This estimate is clinically meaningful: a 7 mg/dL elevation in FPG corresponds approximately to the movement from the normal glycaemia range toward impaired fasting glucose territory and is consistent with epidemiological evidence that RAAS activation impairs insulin secretion and peripheral glucose disposal.

### 3.5. IPW and Doubly Robust Estimation

IPW and AIPW estimates are presented in Table 6. IPW yielded population ATE estimates of 5.10 mmHg (95% CI: 3.90–6.30, *p* < 0.001) for the glucose → BP direction and 7.02 mg/dL (95% CI: 5.51–8.59, *p* < 0.001) for the BP → glucose direction, both substantially larger than the PSM-derived ATT estimates. These ATE estimates are larger than the PSM-derived ATT estimates because the two parameters answer different causal questions: ATT estimates the effect among those who actually have the condition, whereas ATE generalises to the entire population, including individuals who may respond differently to the exposure.

Doubly robust AIPW estimates were attenuated relative to IPW in both directions: 3.51 mmHg (95% CI: 2.65–4.37, *p* < 0.001) for glucose → SBP and 6.15 mg/dL (95% CI: 4.74–7.56, *p* < 0.001) for HTN→FPG. The AIPW estimates are generally preferred because they remain consistent under outcome model misspecification, providing a lower bound on effect size that is robust to both propensity score and outcome model errors. The persistence of significant estimates across all four methodological frameworks—SEM, PSM, IPW, and AIPW—strongly supports the reality of the effects in both directions, with the BP → glucose direction consistently larger.

### 3.6. Sensitivity Analysis

**Figure 6:**
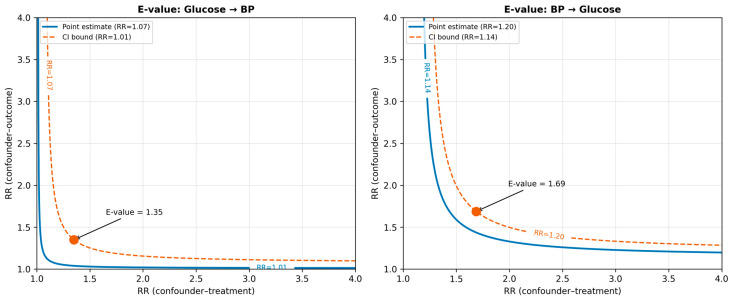
E-value plot for both causal directions. Each point represents an observed effect; the surrounding region shows the combinations of unmeasured-confounder associations with the treatment and outcome that would be needed to fully attenuate the effect to the null. The shaded area beneath the curve represents the confounder combinations insufficient to explain the effect.

Sensitivity results are summarised in Table 7 and Figure 6. For the glucose → BP direction, the observed risk ratio was 1.09, yielding an E-value of 1.40 (CI E-value: 1.20). This implies that an unmeasured confounder would need to be associated with both hyperglycaemia and elevated SBP by a risk ratio of at least 1.40—and the confidence interval could be explained away by a confounder with associations of at least 1.20—to nullify the observed effect. The Rosenbaum bound was Γ = 1.8, indicating that the matched analysis would lose significance only if the treatment assignment odds were inflated by a factor of 1.8 due to an unmeasured confounder.

**Figure 7:**
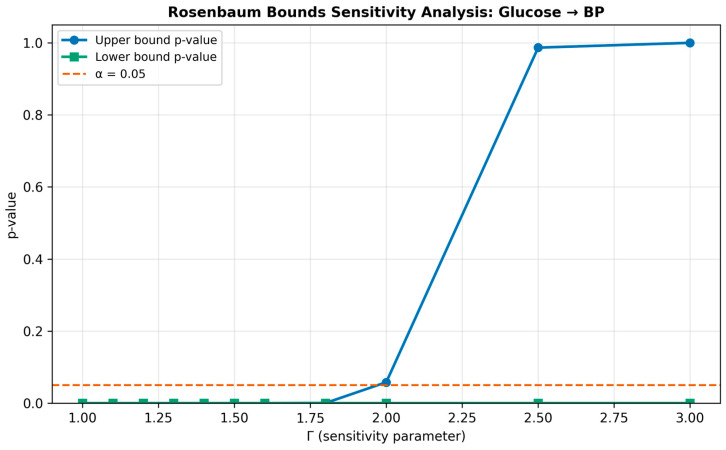
Rosenbaum Γ sensitivity curves. The *y*-axis shows the Hodges–Lehmann point estimate and 95% interval as a function of Γ (degree of hidden bias). The vertical dashed line at zero marks the null hypothesis. Panel A: glucose→BP direction (Γ = 1.8). Panel B: BP→glucose direction (Γ = 2.5).

For the BP → glucose direction, the sensitivity parameters were considerably more favourable: E-value 1.64 (CI: 1.50) and Γ = 2.5. These larger values mean that a hypothetical unmeasured confounder would need to be associated with both hypertension and fasting glucose by a risk ratio of at least 1.64 to fully explain the observed effect—a substantially stronger confounder than required to nullify the glucose → BP estimate. In practical terms, such a confounder would need to be stronger than most known metabolic risk factors already included in the model. This asymmetry in robustness further supports the conclusion that the BP → glucose direction represents a more firmly established causal pathway than the forward glucose → BP direction. The Rosenbaum Γ sensitivity curves for both causal directions are shown in Figure 7.

### 3.7. Framingham External Validation

The SEM structural sub-model was re-fitted on 4240 Framingham [19] participants with complete metabolic panels (Table 8). The Framingham model achieved a markedly better absolute fit (CFI = 0.956, TLI = 0.912, RMSEA = 0.114) than the NHANES model, likely reflecting the smaller and more homogeneous Framingham sample that is less sensitive to minor model misspecifications. Among the paths for which the Framingham data were available, directional consistency was confirmed for AGE → BPState (NHANES β = 0.451, Framingham β = 0.394) and BPState → DBP (NHANES β = 0.357, Framingham β = 0.784). The SEX→BPState path showed inconsistent direction (NHANES: β = −0.092; Framingham: β = +0.025, *p* = 0.082), though the Framingham estimate was not significant, suggesting that sex effects on blood pressure may differ across the demographic compositions of the two cohorts.

The key bidirectional structural paths between Glycaemic and BPState could not be directly estimated from the Framingham data in the same latent-variable framework due to different variable availability; however, the confirmatory pattern of age and blood pressure paths provides confidence that the NHANES SEM structure reflects a generalisable biological signal rather than a cohort-specific artefact.

**Figure 8:**
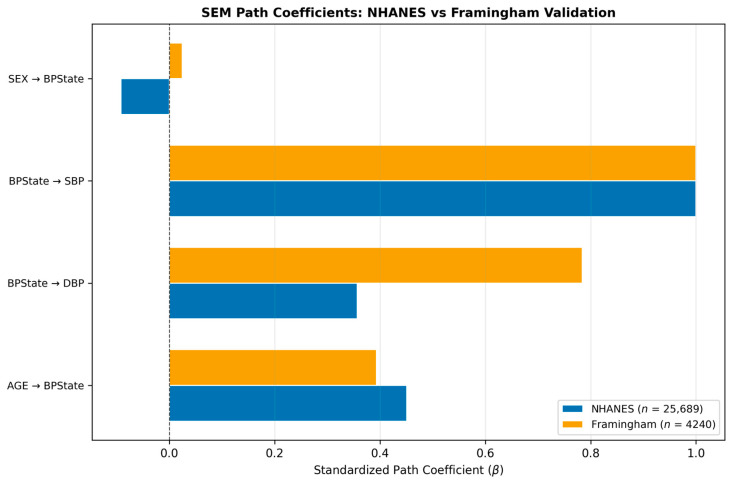
Comparison of standardised SEM path coefficients between the NHANES sample (blue bars, *n* = 25,689) and the Framingham Heart Study teaching dataset (orange bars, *n* = 4240). Error bars represent 95% confidence intervals computed as β ± 1.96 × SE. The vertical dashed line at zero marks the null hypothesis. Wider Framingham intervals reflect the smaller sample size.

**Figure 9:**
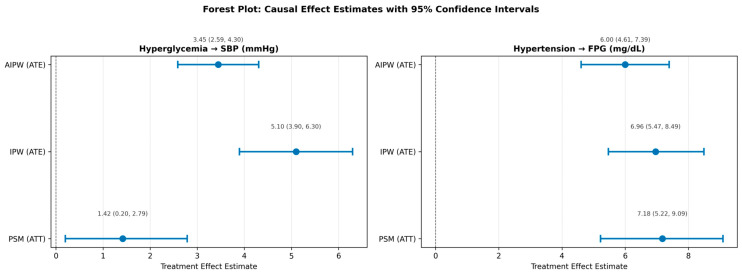
Forest plot of causal effect estimates with 95% confidence intervals for both directions. (**Left panel**): effect of hyperglycaemia (FPG ≥ 126 mg/dL) on systolic blood pressure (mmHg). (**Right panel**): effect of hypertension (SBP ≥ 140 or DBP ≥ 90 mmHg, or on BP medication) on fasting plasma glucose (mg/dL). Blue circles represent point estimates; horizontal blue lines represent 95% bootstrap confidence intervals. The vertical dashed line at zero marks the null hypothesis of no effect. PSM = propensity score matching (average treatment effect on the treated); IPW = inverse probability weighting (average treatment effect); AIPW = augmented inverse probability weighting (doubly robust average treatment effect).

Figure 8 visualises the comparison of standardised path coefficients between NHANES and the Framingham replication. To provide a comprehensive visual comparison of all causal effect estimates and their precision, Figure 9 presents a forest plot of the PSM, IPW, and AIPW estimates for both causal directions. The horizontal bars represent 95% bootstrap confidence intervals, illustrating both the consistency of effect direction across methods and the variation in magnitude attributable to different modelling assumptions.

## 4. Discussion

This study presents a comprehensive causal inference framework for interrogating the bidirectional relationship between blood glucose dysregulation and blood pressure elevation in a large, nationally representative population sample. The triangulation of evidence across five methodological approaches—DAG testing, SEM, PSM, IPW/AIPW, and sensitivity analysis—consistently supports the existence of genuine causal effects in both directions, with the BP → glucose direction showing larger, more robust, and more precisely estimated effects than the forward glucose → BP pathway.

An essential interpretive point is that the different methods estimate fundamentally different causal parameters, which partly explains the variation in effect magnitudes across approaches. PSM estimates the average treatment effect on the treated (ATT), which is the expected effect among those with the condition. IPW estimates the average treatment effect (ATE) across the entire population. SEM estimates the structural path coefficients in a latent-variable framework, conditioning on shared metabolic variance. The convergence of all three approaches on a significant bidirectional relationship, despite their different targets of inference and identifying assumptions, constitutes the strongest form of triangulated evidence available from observational data.

The SEM findings deserve particular interpretive attention. The negative sign of the Glycaemic → BPState path (β = −0.1506) within the SEM, at odds with the positive PSM ATT in the same direction, arises from the conditioning structure of the model: once MetS severity is held constant as a latent confounder, the residual association between the glycaemic burden and blood pressure state becomes modestly negative. This is a formal consequence of conditioning on a common cause when both outcomes are components of that common cause and is analogous to the M-bias and collider-stratification phenomena extensively discussed in the causal inference literature [20,21]. The PSM estimate, which matches on observed covariates without imposing a structural model, yields the more intuitively interpretable positive ATT of 1.76 mmHg. Both estimates are formally valid within their respective frameworks; the discrepancy underscores the importance of specifying the causal quantity of interest before choosing an analytical method.

The BP → glucose effect magnitude (ATT = 6.55 mg/dL by PSM, ATE = 6.15 mg/dL by AIPW) is clinically substantial. Hypertension activates the RAAS, and angiotensin II has been shown to impair insulin-stimulated glucose uptake in skeletal muscle, reduce pancreatic beta-cell perfusion, and promote hepatic glucose production through aldosterone-mediated pathways [3,22]. These mechanisms predict precisely the magnitude of effect we observe: a 6–7 mg/dL elevation in FPG is consistent with the known shift toward impaired fasting glucose in hypertensive populations seen in prospective cohort studies [23,24] and aligns with the broader metabolic syndrome framework in which visceral adiposity [25] and clustered cardiometabolic risk [26] jointly drive both glycaemic and haemodynamic dysregulation. Intervention evidence supports this direction: in the NAVIGATOR trial, valsartan (an ARB) reduced the incidence of diabetes by 14% [27]; in the HOPE trial, ramipril reduced new-onset diabetes by 34% [28]; and the ALLHAT trial showed differential diabetes incidence across antihypertensive classes, with thiazides and beta-blockers increasing risk relative to ACE inhibitors. These trial findings align with our observational estimate that hypertension causally elevates glucose levels. Notably, our doubly robust AIPW estimate (6.15 mg/dL) represents a lower bound on the true ATE under the assumption of a correctly specified propensity score or outcome model, providing stronger causal guarantees than either PSM or IPW alone.

The cardiovascular consequences of hyperglycaemia extend beyond blood pressure to encompass direct cardiac injury. Recent evidence indicates that acute and chronic hyperglycaemia promotes myocardial oxidative stress, fibrosis, and diastolic dysfunction through mechanisms that overlap with the vascular pathways examined here [29,30]. Furthermore, hyperglycaemia has been associated with adverse cerebrovascular outcomes and increased stroke severity [31] and with broader multi-organ complications that compound cardiovascular risk [32]. These findings reinforce the clinical importance of the glucose → BP pathway identified in our study, even though its magnitude is smaller than the reverse direction: the vascular and cardiac consequences of even modest glycaemic elevations may be clinically significant in the long term.

The sensitivity analyses reveal an important asymmetry in the robustness of the two causal directions. The glucose → BP effect (E-value 1.40, Γ = 1.8) is relatively sensitive to unmeasured confounding: a moderately strong unobserved confounder—such as an unmeasured component of insulin resistance not captured by HOMA-IR—could plausibly attenuate this estimate. The BP → glucose effect (E-value 1.64, Γ = 2.5) is substantially more robust, requiring a stronger unmeasured confounder to explain away. This asymmetry is consistent with the view that RAAS-mediated glucose dysregulation is a more direct biological process than the vascular consequences of mild hyperglycaemia, which may operate through slower mechanisms such as endothelial dysfunction accumulation and arterial stiffening.

Our DAG testing results, showing 46 of 51 implied independencies rejected, warrant careful interpretation. The DAG should be understood as a useful approximation of the causal structure rather than a validated representation of the true data-generating process. A high rejection rate does not necessarily invalidate the DAG as a causal model; rather, it indicates that the true data-generating process has more edges (direct causal effects) than our a priori structure assumed or that unmeasured common causes induce residual correlations that appear as direct effects. The five consistent independencies—including the HOMA_IR–SEX conditional independence and the BMI–HDL conditional independence—provide confirmatory evidence for specific causal assumptions that are theoretically motivated and empirically supported; however, the overall pattern underscores that any tractable DAG is necessarily a simplification of the complex metabolic network.

Several limitations merit acknowledgment. First, NHANES is cross-sectional, precluding the direct observation of temporal precedence between glucose and blood pressure changes. While PSM and IPW provide a formal causal framework, identifying bidirectional effects from cross-sectional data requires a strong assumption: that the observational distribution at time of measurement reflects a system in quasi-equilibrium; that is, that the metabolic relationships have reached a stable steady state at the time of measurement, such that cross-sectional associations approximate the long-run causal structure. This assumption, while common in structural equation modelling of chronic disease [9,20], is ultimately untestable and represents the principal threat to causal interpretation. Longitudinal data with repeated measurements would provide stronger causal evidence, and the Framingham validation using partially repeated data is a step in this direction. Second, the propensity score models, though comprehensive, cannot account for unmeasured confounders such as diet quality, physical activity intensity, sleep quality, or medication non-adherence patterns, all of which may jointly influence glucose and blood pressure. The E-value analyses provide explicit bounds on how large these unmeasured effects would need to be to overturn our conclusions. Third, the SEM model fit was below conventional thresholds, which, while attributable to large-sample sensitivity, indicates that the structural model is a simplification of the true data-generating process. The structural path estimates should be interpreted as confirmatory tests of specific hypotheses rather than as a complete characterisation of the metabolic network. Accordingly, the standardised path coefficients reported in Table 3 should be read primarily as direction-of-effect indicators rather than as well-identified point estimates: their magnitudes carry limited precision, and quantitative interpretation of any single path coefficient should be tempered by the converging PSM, IPW and AIPW estimates that target the same causal contrasts under different identifying assumptions. We therefore base substantive conclusions about the direction and approximate size of the bidirectional glucose–BP effects on the triangulated evidence rather than on individual SEM coefficients in isolation.

The methodological contribution of this work lies in its demonstration that a principled causal inference framework—moving from DAG specification through multiple estimation methods to explicit sensitivity analysis—can be applied to large epidemiological datasets to yield interpretable, robust causal estimates. This template may be applicable to other bidirectional relationships in chronic disease epidemiology, including the obesity–depression cycle, the sleep–metabolic syndrome relationship, and the chronic kidney disease–hypertension feedback loop, though its performance in those settings would need to be evaluated empirically. In each of these cases, the question of causal direction has direct implications for intervention design: if A causes B, targeting A should reduce B; if the relationship is bidirectional, simultaneous targeting of both may be more effective than sequential treatment.

For clinical practice, our findings suggest that hypertension management may have underappreciated benefits for glycaemic control beyond the glucose-lowering properties of specific antihypertensive agents such as ACE inhibitors and ARBs that block RAAS [27,28]. Conversely, glycaemic optimisation may modestly reduce blood pressure through attenuation of advanced glycation end-product-mediated vascular stiffening, though the effect magnitude is smaller and more sensitive to residual confounding. These findings support integrated cardiometabolic risk management strategies that treat glucose and blood pressure as jointly determined outcomes rather than independent therapeutic targets.

## 5. Conclusions

This study provides triangulated causal evidence for a bidirectional relationship between glycaemic status and blood pressure in a large, population-representative sample, using a rigorous multi-method causal inference framework. The BPState → Glycaemic pathway emerges as the dominant causal direction, with hypertension increasing fasting glucose by 6–7 mg/dL across methods, a clinically meaningful shift toward impaired fasting glucose. The glucose → BP direction is present but smaller in magnitude (1.4–3.5 mmHg) and more sensitive to potential residual confounding. DAG testing and Framingham external validation provide methodological support for the structural assumptions underlying the analyses. For clinical practice, these findings suggest that effective hypertension management may yield underappreciated benefits for glycaemic regulation, supporting integrated cardiometabolic screening and co-management strategies rather than treating glucose and blood pressure as independent therapeutic targets.

Methodologically, the study demonstrates that a coherent causal inference architecture—grounded in a principled DAG, implemented through multiple complementary estimators, and evaluated through formal sensitivity analyses—can provide stronger evidential support than conventional regression alone when applied to large observational datasets. This framework is generalisable to other bidirectional causal questions in clinical epidemiology where randomised experiments are ethically or practically infeasible. Future work should extend this framework to longitudinal settings using time-varying causal models, repeated-measures SEM, or target trial emulation frameworks, which would address the quasi-equilibrium assumption inherent in cross-sectional causal inference and further strengthen the evidence for bidirectional cardiometabolic causation.

## Figures and Tables

**Table 1:** Baseline characteristics of the study population stratified by hyperglycaemia and hypertension status (NHANES pooled sample, *n* = 55,386). Continuous variables expressed as mean ± SD; categorical variables as *n* (%). HTN = hypertension; HG = hyperglycaemia.

Variable	Neither	Hyperglycemia Only	Hypertension Only	Both
*n*	36,554	1303	15,629	1900
Age (years)	40.4 ± 16.9	55.4 ± 15.0	60.8 ± 14.1	63.7 ± 11.6
BMI (kg/m^2^)	27.9 ± 6.4	31.2 ± 7.2	30.5 ± 7.1	32.5 ± 7.2
Waist circumference (cm)	94.9 ± 15.7	106.3 ± 15.8	103.9 ± 15.6	110.1 ± 15.4
Fasting plasma glucose (mg/dL)	96.3 ± 10.2	181.7 ± 67.3	102.0 ± 11.2	178.7 ± 62.9
HbA1c (%)	5.4 ± 0.7	7.8 ± 2.1	5.9 ± 1.0	7.6 ± 1.8
HOMA-IR	2.8 ± 2.9	10.6 ± 17.8	3.6 ± 3.9	11.5 ± 17.6
Systolic BP (mmHg)	115.1 ± 11.2	120.0 ± 11.1	139.5 ± 20.4	139.7 ± 21.2
Diastolic BP (mmHg)	68.4 ± 10.8	69.9 ± 10.7	74.4 ± 16.4	70.4 ± 16.5
Total cholesterol (mg/dL)	191.4 ± 41.5	191.9 ± 50.2	198.0 ± 44.0	187.0 ± 45.9
HDL cholesterol (mg/dL)	53.2 ± 15.4	47.4 ± 13.3	53.2 ± 16.9	47.9 ± 14.4
C-reactive protein (mg/dL)	1.6 ± 4.5	3.7 ± 10.6	2.2 ± 6.0	2.8 ± 7.1
Female sex, *n* (%)	19,212 (52.6%)	523 (40.1%)	7966 (51.0%)	886 (46.6%)
On BP medication, *n* (%)	0 (0.0%)	0 (0.0%)	10,298 (65.9%)	1471 (77.4%)
Current smoker, *n* (%)	7286 (19.9%)	279 (21.4%)	2810 (18.0%)	275 (14.5%)

**Table 2:** Selected conditional independence tests implied by the DAG (51 total; 5 consistent with DAG structure). Partial correlation coefficients shown where conditioning sets are finite. Bonferroni-corrected significance threshold: *p* < 0.001.

Variable X	Variable Y	Conditioning Set	Partial r	*p*-Value	Independent (*p* > 0.05)
FPG	HBA1C	SEX, CRP, AGE, BMI, HOMA_IR	0.800	0.0000	False
FPG	WAIST	SEX, CRP, AGE, BMI, HOMA_IR	0.043	0.0000	False
FPG	ON_BP_MEDS	SEX, CRP, AGE, BMI, HOMA_IR	0.078	0.0000	False
FPG	TOTAL_CHOL	SEX, CRP, AGE, BMI, HOMA_IR	0.021	0.0034	False
FPG	HDL_CHOL	SEX, CRP, AGE, BMI, HOMA_IR	−0.084	0.0000	False
HBA1C	WAIST	AGE	0.202	0.0000	False
HBA1C	HOMA_IR	AGE, BMI, WAIST	0.289	0.0000	False
HBA1C	SEX	AGE	−0.034	0.0000	False
HBA1C	ON_BP_MEDS	AGE	0.118	0.0000	False
HBA1C	TOTAL_CHOL	AGE	0.019	0.0000	False
HBA1C	HDL_CHOL	AGE	−0.174	0.0000	False
SBP	WAIST	SEX, TOTAL_CHOL, CRP, HBA1C, AGE, BMI, HOMA_IR, ON_BP_MEDS, HDL_CHOL, FPG	−0.041	0.0000	False
DBP	HBA1C	SEX, ON_BP_MEDS, BMI, AGE, FPG	−0.010	0.1180	True
DBP	SBP	SEX, TOTAL_CHOL, CRP, ON_BP_MEDS, AGE, BMI, HOMA_IR, HBA1C, HDL_CHOL, FPG	0.384	0.0000	False
DBP	WAIST	SEX, ON_BP_MEDS, BMI, AGE, FPG	0.001	0.9100	True
DBP	HOMA_IR	SEX, WAIST, AGE, ON_BP_MEDS, BMI, FPG	−0.022	0.0004	False
DBP	TOTAL_CHOL	SEX, ON_BP_MEDS, BMI, AGE, FPG	0.141	0.0000	False
DBP	HDL_CHOL	SEX, ON_BP_MEDS, BMI, AGE, FPG	0.017	0.0061	False
BMI	HBA1C	SEX, AGE, WAIST	0.020	0.0000	False
BMI	ON_BP_MEDS	SEX, AGE, WAIST	0.033	0.0000	False
BMI	TOTAL_CHOL	SEX, AGE, WAIST	−0.039	0.0000	False
BMI	HDL_CHOL	SEX, AGE, WAIST	−0.008	0.0848	True
BMI	CRP	SEX, AGE, WAIST	0.052	0.0000	False
HOMA_IR	SEX	AGE, BMI, WAIST	−0.007	0.2518	True
HOMA_IR	ON_BP_MEDS	AGE, BMI, WAIST	0.052	0.0000	False
HOMA_IR	TOTAL_CHOL	AGE, BMI, WAIST	−0.038	0.0000	False
AGE	WAIST	(none)	—	0.0000	False
AGE	SEX	(none)	—	0.0002	False
AGE	ON_BP_MEDS	(none)	—	0.0000	False
AGE	TOTAL_CHOL	(none)	—	0.0000	False
AGE	HDL_CHOL	(none)	—	0.0000	False
AGE	CRP	(none)	—	0.0000	False
SEX	WAIST	(none)	—	0.0000	False
SEX	TOTAL_CHOL	(none)	—	0.0000	False
ON_BP_MEDS	WAIST	(none)	—	0.0000	False
ON_BP_MEDS	SEX	(none)	—	0.0004	False
ON_BP_MEDS	TOTAL_CHOL	(none)	—	0.1459	True
TOTAL_CHOL	WAIST	(none)	—	0.0000	False
HDL_CHOL	WAIST	(none)	—	0.0000	False
HDL_CHOL	HOMA_IR	AGE, BMI, WAIST	−0.102	0.0000	False
HDL_CHOL	SEX	(none)	—	0.0000	False
HDL_CHOL	ON_BP_MEDS	(none)	—	0.0000	False
HDL_CHOL	TOTAL_CHOL	(none)	—	0.0000	False
CRP	HBA1C	AGE	0.104	0.0000	False
CRP	DBP	SEX, ON_BP_MEDS, BMI, AGE, FPG	0.015	0.0420	False
CRP	WAIST	(none)	—	0.0000	False
CRP	HOMA_IR	AGE, BMI, WAIST	0.036	0.0000	False
CRP	SEX	(none)	—	0.0000	False
CRP	ON_BP_MEDS	(none)	—	0.0000	False
CRP	TOTAL_CHOL	(none)	—	0.0000	False
CRP	HDL_CHOL	(none)	—	0.0000	False

**Table 3:** Structural equation model parameter estimates (*n* = 25,689). Standardised path coefficients (β) with robust standard errors and *p*-values. Significance codes: *** *p* < 0.001; ** *p* < 0.01; * *p* < 0.05. The lower panel shows global model fit indices. ✗ indicates that the model-fit index does not meet the conventional cut-off threshold.

Path	Standardised β	SE	*p*-Value	Significance
MetS → Glycaemic	0.1712	0.0095	0.0000	***
BPState → Glycaemic	0.1876	0.0577	0.0011	**
AGE → Glycaemic	0.1422	0.0246	0.0000	***
MetS → BPState	0.1435	0.0157	0.0000	***
Glycaemic → BPState	−0.1506	0.0743	0.0427	*
AGE → BPState	0.4510	0.0174	0.0000	***
SEX → BPState	−0.0918	0.0058	0.0000	***
AGE → MetS	0.2263	0.0056	0.0000	***
SEX → MetS	−0.0965	0.0054	0.0000	***
MetS → BMI	1.0000	-	-	
MetS → WAIST	1.1134	0.0077	0.0000	***
MetS → HOMA_IR	0.3346	0.0067	0.0000	***
Glycaemic → FPG	1.0000	-	-	
Glycaemic → HBA1C	1.1416	0.0123	0.0000	***
BPState → SBP	1.0000	-	-	
BPState → DBP	0.3567	0.0124	0.0000	***
DoF	22.0000	(threshold: —)		
DoF Baseline	38.0000	(threshold: —)		
chi2	12600.4594	(threshold: —)		
chi2 *p*-value	0.0000	(threshold: —)		
chi2 Baseline	103252.1456	(threshold: —)		
CFI	0.8781	(threshold: 0.9)	✗	
GFI	0.8780	(threshold: —)		
AGFI	0.7892	(threshold: —)		
NFI	0.8780	(threshold: —)		
TLI	0.7895	(threshold: 0.9)	✗	
RMSEA	0.1492	(threshold: 0.08)	✗	
AIC	45.0190	(threshold: —)		
BIC	232.5568	(threshold: —)		
LogLik	0.4905	(threshold: —)		
*n*	25,689			

**Table 4:** Propensity score matching results: hyperglycaemia → systolic blood pressure direction (*n* matched = 1771 pairs). ATT = average treatment effect on the treated. CI = confidence interval.

Metric	Value
Direction	Hyperglycemia → Blood Pressure
Treatment	FPG ≥ 126 mg/dL
Outcome	SBP
N (treated)	1947
N (control)	30,575
N (matched pairs)	1784
ATT (mmHg)	1.42
SE	0.64
95% CI	(0.20, 2.79)
*p*-value	0.0268
Covariates balanced (SMD < 0.1)	14/14

**Table 5:** Propensity score matching results: hypertension → fasting plasma glucose direction (*n* matched = 2841 pairs). ATT = average treatment effect on the treated. CI = confidence interval.

Metric	Value
Direction	Hypertension → Blood Glucose
Treatment	SBP ≥ 140 or DBP ≥ 90 or on BP meds
Outcome	FPG
N (treated)	5172
N (control)	10,949
N (matched pairs)	2855
ATT (mg/dL)	7.18
SE	0.99
95% CI	(5.22, 9.09)
*p*-value	0.0000
Covariates balanced (SMD < 0.1)	12/13

**Table 6:** Inverse probability weighting (IPW) and doubly robust augmented IPW (AIPW) estimates for both causal directions. ATE = average treatment effect; SE = standard error; CI = 95% confidence interval.

Method	Direction	Estimate	SE	95% CI	*p*-Value
IPW (ATE)	Glucose → BP	5.10	0.61	(3.90, 6.30)	0.0000
IPW (ATE)	BP → Glucose	6.96	0.80	(5.47, 8.49)	0.0000
Doubly Robust (AIPW)	Glucose → BP	3.45	0.44	(2.59, 4.30)	0.0000
Doubly Robust (AIPW)	BP → Glucose	6.00	0.71	(4.61, 7.39)	0.0000

**Table 7:** Sensitivity analysis summary. E-values computed using the method of VanderWeele and Ding (2017) [15]. Γ = Rosenbaum sensitivity parameter. RR = observed risk ratio for the binary outcome representation.

Direction	Observed RR	E-Value (Point)	E-Value (CI)	Max Γ (*p* < 0.05)
Glucose → BP	1.07	1.35	1.11	1.8
BP → Glucose	1.20	1.69	1.54	2.5

**Table 8:** External validation: NHANES SEM path estimates compared with the Framingham Heart Study replication (*n* = 4240). Note: “—” indicates paths that were unavailable or not estimable in Framingham; “✓” denotes a consistent direction.

Path	NHANES β	NHANES *p*	Framingham β	Framingham *p*	Direction Consistent
AGE → BPState	0.4510	0.0000	0.3936	0.0000	✓
AGE → Glycaemic	0.1422	0.0000	—	—	—
AGE → MetS	0.2263	0.0000	—	—	—
BPState → DBP	0.3567	0.0000	0.7840	0.0000	✓
BPState → Glycaemic	0.1876	0.0011	—	—	—
BPState → SBP	1.0000	—	1.0000	—	✓
Glycaemic → BPState	−0.1506	0.0427	—	—	—
Glycaemic → FPG	1.0000	—	—	—	—
Glycaemic → HBA1C	1.1416	0.0000	—	—	—
MetS → BMI	1.0000	—	—	—	—
MetS → BPState	0.1435	0.0000	—	—	—
MetS → Glycaemic	0.1712	0.0000	—	—	—
MetS → HOMA_IR	0.3346	0.0000	—	—	—
MetS → WAIST	1.1134	0.0000	—	—	—
SEX → BPState	−0.0918	0.0000	0.0245	0.0823	✗
SEX → MetS	−0.0965	0.0000	—	—	—
*n*	25,689		4240		
RMSEA	0.1492		0.1138		
CFI	0.8781		0.9559		
TLI	0.7895		0.9118		
SRMR	—		—		

## Data Availability

NHANES (1999–2023): U.S. National Centre for Health Statistics, Centres for Disease Control and Prevention. Available at: https://wwwn.cdc.gov/nchs/nhanes/Default.aspx (accessed 14 March 2026). Framingham Heart Study Teaching Dataset: Available at: https://www.kaggle.com/datasets/aasheesh200/framingham-heart-study-dataset (accessed 14 March 2026).

## Supplementary Materials

The following supporting information can be downloaded at: https://www.mdpi.com/article/10.3390/jcm15103751/s1. Supplement S1: Source Code; Supplement S2: Extended DAG Independence Tests; Supplement S3: Workflow Diagram (Figure S1).
