# Supplementary material for: Bidirectional Associations Between Blood Glucose and Blood Pressure: A Data-Driven Causal Analysis Using Structural Equation Modelling and Granger Causality on NHANES Longitudinal Data
Source: J Clin Med. 2026 May 13;15(10):3751. doi: 10.3390/jcm15103751 (PMC13207677; doi:10.3390/jcm15103751)
Supplement: Supplementary file 1 [file jcm-15-03751-s001.zip › Supplement_S1_Source_Code.pdf]

Supplementary Material S1: Python Source Code

Bidirectional Causal Relationship Between Blood Glucose and Blood Pressure: A Causal Inference Analysis of NHANES Data

This supplement contains the complete Python source code used in the analysis. All code is presented verbatim with line numbers. The codebase implements causal inference methods including Structural Equation Modeling (SEM), Propensity Score Matching (PSM), Inverse Probability Weighting (IPW), Augmented IPW (AIPW), E-value sensitivity analysis, and Rosenbaum bounds.

File: src/config.py

Lines: 197

```python
"""Configuration for the Bidirectional Causal Inference analysis pipeline.

This article uses the same NHANES data infrastructure as Article 1 (BP_CL_260314)
but applies completely different analytical methods (SEM, PSM, IPW, DAG, E-values).
"""

from dataclasses import dataclass, field
from pathlib import Path
from typing import Dict, List, Tuple

BASE_DIR = Path(__file__).resolve().parent.parent
DATA_DIR = BASE_DIR / "data"
RAW_DIR = DATA_DIR / "nhanes_raw"
PROCESSED_DIR = DATA_DIR / "nhanes_processed"
FRAMINGHAM_DIR = DATA_DIR / "framingham"
RESULTS_DIR = BASE_DIR / "results"
FIGURES_DIR = RESULTS_DIR / "figures"
TABLES_DIR = RESULTS_DIR / "tables"
LOGS_DIR = RESULTS_DIR / "logs"

# Article 1 directory (for shared NHANES data loading)
ARTICLE1_DIR = BASE_DIR.parent / "BP_CL_260314"
ARTICLE1_DATA_DIR = ARTICLE1_DIR / "data" / "nhanes_processed"

RANDOM_SEED = 42

# --- Propensity Score Matching Configuration ---

# Direction 1: Glucose -> BP
PSM_GLUCOSE_TO_BP = {
    "treatment": "hyperglycemia",  # FPG >= 126 mg/dL
    "outcome_continuous": "SBP",
    "outcome_binary": "HTN",
    "covariates": [
        "AGE", "SEX", "BMI", "WAIST", "RACE_ETH_encoded",
        "EDUCATION", "INCOME_RATIO", "ON_BP_MEDS",
        "TOTAL_CHOL", "HDL_CHOL", "CRP", "SMOKING",
        "PHYSICAL_ACTIVITY", "ALCOHOL",
    ],
    "caliper": 0.2,  # in SD of logit propensity score
    "matching_ratio": 1,  # 1:1 matching
}

# Direction 2: BP -> Glucose
PSM_BP_TO_GLUCOSE = {
    "treatment": "HTN",  # SBP >= 140 or DBP >= 90 or on BP meds
    "outcome_continuous": "FPG",
    "outcome_binary": "hyperglycemia",
    "covariates": [
        "AGE", "SEX", "BMI", "WAIST", "RACE_ETH_encoded",
        "EDUCATION", "INCOME_RATIO",
        "TOTAL_CHOL", "HDL_CHOL", "CRP", "SMOKING",
        "PHYSICAL_ACTIVITY", "ALCOHOL",
    ],
    "caliper": 0.2,
    "matching_ratio": 1,
}

# SMD threshold for acceptable balance
SMD_THRESHOLD = 0.1

# IPW weight trimming percentiles
IPW_TRIM_LOWER = 1  # percentile
IPW_TRIM_UPPER = 99  # percentile

# --- SEM Configuration ---

SEM_MEASUREMENT_MODEL = {
    "MetS": ["BMI", "WAIST", "HOMA_IR"],  # Metabolic Syndrome latent
    "Glycemic": ["FPG", "HBA1C"],  # Glycemic State latent
    "BPState": ["SBP", "DBP"],  # Blood Pressure State latent
}

SEM_STRUCTURAL_PATHS = [
    ("MetS", "Glycemic"),      # Metabolic syndrome -> glycemic state
    ("MetS", "BPState"),       # Metabolic syndrome -> BP state
    ("Glycemic", "BPState"),   # Direct glucose -> BP path (of interest)
    ("BPState", "Glycemic"),   # Reciprocal BP -> glucose path (of interest)
    ("AGE", "MetS"),           # Age -> metabolic syndrome
```

```
    ("AGE", "Glycemic"),       # Age -> glycemic state
    ("AGE", "BPState"),        # Age -> BP state
    ("SEX", "MetS"),           # Sex -> metabolic syndrome
    ("SEX", "BPState"),        # Sex -> BP state
]

SEM_FIT_THRESHOLDS = {
    "RMSEA": 0.08,   # acceptable if < 0.08, good if < 0.06
    "CFI": 0.90,     # acceptable if > 0.90, good if > 0.95
    "TLI": 0.90,     # acceptable if > 0.90
    "SRMR": 0.08,    # acceptable if < 0.08
}

# --- DAG Configuration ---

DAG_NODES = [
    "FPG", "HBA1C", "SBP", "DBP",
    "BMI", "WAIST", "HOMA_IR",
    "AGE", "SEX",
    "ON_BP_MEDS", "TOTAL_CHOL", "HDL_CHOL", "CRP",
]

DAG_EDGES = [
    # Metabolic syndrome cluster
    ("BMI", "HOMA_IR"),
    ("BMI", "FPG"),
    ("BMI", "SBP"),
    ("BMI", "DBP"),
    ("WAIST", "HOMA_IR"),
    ("WAIST", "BMI"),
    # Insulin resistance pathway
    ("HOMA_IR", "FPG"),
    ("HOMA_IR", "SBP"),
    # Glucose -> BP (hypothesis 1)
    ("FPG", "SBP"),
    ("FPG", "DBP"),
    ("HBA1C", "SBP"),
    # BP -> Glucose (hypothesis 2) -- tested in bidirectional SEM
    # ("SBP", "FPG"),  # NOT in DAG (tested via SEM)
    # Demographics
    ("AGE", "FPG"),
    ("AGE", "HBA1C"),
    ("AGE", "SBP"),
    ("AGE", "DBP"),
    ("AGE", "BMI"),
    ("AGE", "HOMA_IR"),
    ("SEX", "BMI"),
    ("SEX", "SBP"),
    ("SEX", "DBP"),
    ("SEX", "FPG"),
    # Lipids and inflammation
    ("TOTAL_CHOL", "SBP"),
    ("HDL_CHOL", "SBP"),
    ("CRP", "SBP"),
    ("CRP", "FPG"),
    # Medication
    ("ON_BP_MEDS", "SBP"),
    ("ON_BP_MEDS", "DBP"),
]

# --- E-value Configuration ---

EVALUE_ALPHA = 0.05  # significance level

# --- Analysis Parameters ---

AGE_MIN = 18
AGE_MAX = 80
FPG_THRESHOLD = 126  # mg/dL for hyperglycemia
SBP_THRESHOLD = 140  # mmHg for hypertension
DBP_THRESHOLD = 90   # mmHg for hypertension

# Bootstrap parameters
N_BOOTSTRAP = 1000
BOOTSTRAP_CI = 0.95

# --- Visualization ---

FIG_DPI = 300
FIG_FORMAT_RASTER = "png"
FIG_FORMAT_VECTOR = "pdf"

COLORBLIND_PALETTE = [
    "#0072B2",  # blue
```

```
    "#E69F00",  # orange
    "#009E73",  # green
    "#CC79A7",  # pink
    "#D55E00",  # red-orange
    "#56B4E9",  # light blue
    "#F0E442",  # yellow
    "#000000",  # black
    "#999999",  # gray
    "#882255",  # wine
]

# Figures plan
FIGURES_PLAN = {
    "fig1": "DAG: Directed Acyclic Graph for glucose <-> BP causal structure",
    "fig2": "SEM path diagram with standardized coefficients",
    "fig3": "Love plot: covariate balance before/after PSM (glucose -> BP)",
    "fig4": "Love plot: covariate balance before/after PSM (BP -> glucose)",
    "fig5": "E-value contour plots for both causal directions",
    "fig6": "SEM path comparison: NHANES vs Framingham coefficients",
    "fig7": "Propensity score distribution overlap (treated vs control)",
    "fig8": "Rosenbaum bounds sensitivity analysis at varying ?",
}

# Tables plan
TABLES_PLAN = {
    "table1": "Baseline characteristics by hyperglycemia and hypertension status",
    "table2": "DAG conditional independence test results",
    "table3": "SEM standardized path coefficients with fit indices",
    "table4": "PSM results: effect of hyperglycemia on BP (ATT)",
    "table5": "IPW and doubly robust estimates (both directions)",
    "table6": "PSM results: effect of hypertension on glucose (ATT)",
    "table7": "E-values and Rosenbaum ? for all estimates",
    "table8": "Framingham validation: SEM coefficients comparison",
}
```

File: src/data_loader.py

Lines: 325

```
"""Data loading for bidirectional causal inference analysis.

Loads NHANES data (via Article 1's infrastructure or standalone)
and Framingham Heart Study teaching dataset.
"""

import logging
import sys
from pathlib import Path
from typing import Optional

import numpy as np
import pandas as pd

from .config import (
    ARTICLE1_DIR, ARTICLE1_DATA_DIR, RAW_DIR, PROCESSED_DIR,
    FRAMINGHAM_DIR, FPG_THRESHOLD, SBP_THRESHOLD, DBP_THRESHOLD,
    AGE_MIN, AGE_MAX,
)

logger = logging.getLogger(__name__)


def load_nhanes_from_article1(cache: bool = True) -> Optional[pd.DataFrame]:
    """Load NHANES data using Article 1's data loader.

    This avoids duplicating the complex NHANES harmonization code.
    Falls back to loading from a cached parquet file if Article 1's
    code is not available.
    """
    cache_file = PROCESSED_DIR / "nhanes_causal_ready.parquet"

    # Try loading from our own processed cache first
    if cache and cache_file.exists():
        logger.info(f"Loading cached causal-ready data from {cache_file}")
        return pd.read_parquet(cache_file)

    # Load raw merged data from Article 1's cache or our own
    raw_df = None
    for candidate in [
        ARTICLE1_DATA_DIR / "nhanes_merged_all.parquet",
        PROCESSED_DIR / "nhanes_merged_all.parquet",
    ]:
        if candidate.exists():
            logger.info(f"Loading NHANES merged data from: {candidate}")
            raw_df = pd.read_parquet(candidate)
            break

    if raw_df is None:
        logger.error(
            "No NHANES data available. Either:\n"
            "  1. Run Article 1's download_nhanes.py + run_analysis.py first, OR\n"
            "  2. Place nhanes_merged_all.parquet in data/nhanes_processed/"
        )
        return None

    # Derive all variables needed for causal analysis
    df = _derive_nhanes_variables(raw_df)

    # Merge smoking data if available
    smoking_file = PROCESSED_DIR / "nhanes_smoking.parquet"
    if smoking_file.exists():
        smq = pd.read_parquet(smoking_file)
        df = df.merge(smq[["SEQN", "CURRENT_SMOKER", "EVER_SMOKER"]],
                      on="SEQN", how="left")
        df["SMOKING"] = df["CURRENT_SMOKER"].fillna(0).astype(int)
        df["EVER_SMOKER"] = df["EVER_SMOKER"].fillna(0).astype(int)
        logger.info(f"Merged smoking data: {df['SMOKING'].sum():,} current smokers "
                    f"({100*df['SMOKING'].mean():.1f}%)")
    else:
        logger.warning("Smoking data not found -- run download to fetch SMQ data")

    # Save processed cache
    PROCESSED_DIR.mkdir(parents=True, exist_ok=True)
    df.to_parquet(cache_file, index=False)
    logger.info(f"Cached causal-ready data: {len(df):,} records")

    return df

```

```python

def _derive_nhanes_variables(df: pd.DataFrame) -> pd.DataFrame:
    """Derive all analysis variables from raw NHANES merged data.

    Creates: HOMA_IR, HTN, ON_BP_MEDS, hyperglycemia, RACE_ETH,
    SEX_LABEL, DM_STATUS, AGE_GROUP, BMI_CAT, PP, MAP, etc.
    """
    df = df.copy()

    # Age filter
    df = df[(df["AGE"] >= AGE_MIN) & (df["AGE"] <= AGE_MAX)]

    # Require SBP
    df = df[df["SBP"].notna()]

    # Require at least FPG or HBA1C
    has_glucose = df["FPG"].notna()
    if "HBA1C" in df.columns:
        has_glucose = has_glucose | df["HBA1C"].notna()
    df = df[has_glucose]

    # Require positive survey weight
    if "WEIGHT_MEC" in df.columns:
        df = df[df["WEIGHT_MEC"].notna() & (df["WEIGHT_MEC"] > 0)]

    # HOMA-IR
    if "FPG" in df.columns and "INSULIN" in df.columns:
        mask = df["FPG"].notna() & df["INSULIN"].notna() & (df["INSULIN"] > 0)
        df["HOMA_IR"] = np.where(mask, df["FPG"] * df["INSULIN"] / 405.0, np.nan)
        logger.info(f"HOMA-IR computed for {mask.sum():,} records")

    # Sex label
    if "SEX" in df.columns:
        df["SEX_LABEL"] = df["SEX"].map({1: "Male", 2: "Female"})

    # BP medication
    if "TAKING_BP_MEDS" in df.columns:
        df["ON_BP_MEDS"] = (df["TAKING_BP_MEDS"] == 1).astype(int)
    else:
        df["ON_BP_MEDS"] = 0

    # Hypertension
    htn_mask = df["SBP"] >= SBP_THRESHOLD
    if "DBP" in df.columns:
        htn_mask = htn_mask | (df["DBP"] >= DBP_THRESHOLD)
    htn_mask = htn_mask | (df["ON_BP_MEDS"] == 1)
    df["HTN"] = htn_mask.astype(int)

    # Hyperglycemia
    if "FPG" in df.columns:
        df["hyperglycemia"] = (df["FPG"] >= FPG_THRESHOLD).astype(int)

    # Race/ethnicity harmonization
    for race_col in ["RACE", "RACE_OLD"]:
        if race_col in df.columns:
            race_map = {
                1: "Mexican American",
                2: "Other Hispanic",
                3: "Non-Hispanic White",
                4: "Non-Hispanic Black",
                5: "Other/Multiracial",
                6: "Non-Hispanic Asian",
                7: "Other/Multiracial",
            }
            df["RACE_ETH"] = df[race_col].map(race_map)
            break

    # Pulse pressure and MAP
    if "SBP" in df.columns and "DBP" in df.columns:
        df["PP"] = df["SBP"] - df["DBP"]
        df["MAP"] = df["DBP"] + (df["SBP"] - df["DBP"]) / 3.0

    # Diabetes status
    if "FPG" in df.columns:
        conditions = [
            df["FPG"] < 100,
            df["FPG"].between(100, 126, inclusive="left"),
            df["FPG"] >= 126,
        ]
        df["DM_STATUS"] = np.select(conditions, ["Normal", "Prediabetes", "Diabetes"],
                                    default="Unknown")

    # Survey weight adjustment for multi-cycle
    if "WEIGHT_MEC" in df.columns:
```

```
        n_cycles = df["CYCLE"].nunique()
        if n_cycles > 0:
            df["WEIGHT_COMBINED"] = df["WEIGHT_MEC"] / n_cycles
    if "WEIGHT_FASTING" in df.columns:
        n_cycles = df["CYCLE"].nunique()
        if n_cycles > 0:
            df["WEIGHT_FASTING_COMBINED"] = df["WEIGHT_FASTING"] / n_cycles

    logger.info(f"Derived variables complete: {len(df):,} records, {len(df.columns)} columns")
    logger.info(f"  Hyperglycemia: {df['hyperglycemia'].sum():,}")
    logger.info(f"  HTN: {df['HTN'].sum():,}")
    logger.info(f"  HOMA_IR available: {df['HOMA_IR'].notna().sum():,}")

    return df


def load_framingham(filepath: Optional[Path] = None) -> Optional[pd.DataFrame]:
    """Load and prepare the Framingham Heart Study teaching dataset.

    Expected columns: male, age, education, currentSmoker, cigsPerDay,
    BPMeds, prevalentStroke, prevalentHyp, diabetes, totChol, sysBP,
    diaBP, BMI, heartRate, glucose.
    """
    if filepath is None:
        # Try common locations
        candidates = [
            FRAMINGHAM_DIR / "framingham.csv",
            FRAMINGHAM_DIR / "framingham_heart_study.csv",
        ]
        for c in candidates:
            if c.exists():
                filepath = c
                break

    if filepath is None or not filepath.exists():
        logger.warning(
            f"Framingham dataset not found. Expected in: {FRAMINGHAM_DIR}/\n"
            "Download from: https://www.kaggle.com/datasets/aasheesh200/"
            "framingham-heart-study-dataset"
        )
        return None

    df = pd.read_csv(filepath)
    logger.info(f"Loaded Framingham dataset: {len(df)} records, {len(df.columns)} columns")

    # Harmonize column names to match NHANES convention
    rename_map = {
        "male": "SEX_MALE",
        "age": "AGE",
        "education": "EDUCATION",
        "currentSmoker": "SMOKING",
        "cigsPerDay": "CIGS_PER_DAY",
        "BPMeds": "ON_BP_MEDS",
        "prevalentStroke": "STROKE",
        "prevalentHyp": "PREVALENT_HTN",
        "diabetes": "DM_STATUS_BINARY",
        "totChol": "TOTAL_CHOL",
        "sysBP": "SBP",
        "diaBP": "DBP",
        "BMI": "BMI",
        "heartRate": "HEART_RATE",
        "glucose": "FPG",
    }
    df = df.rename(columns=rename_map)

    # Create derived variables matching NHANES
    df["SEX"] = np.where(df["SEX_MALE"] == 1, 1, 2)  # 1=Male, 2=Female (NHANES coding)

    # Hyperglycemia and hypertension flags
    df["hyperglycemia"] = (df["FPG"] >= FPG_THRESHOLD).astype(int)
    df["HTN"] = (
        (df["SBP"] >= SBP_THRESHOLD) |
        (df["DBP"] >= DBP_THRESHOLD) |
        (df["ON_BP_MEDS"] == 1)
    ).astype(int)

    # Pulse pressure and MAP
    df["PP"] = df["SBP"] - df["DBP"]
    df["MAP"] = df["DBP"] + (df["SBP"] - df["DBP"]) / 3.0

    logger.info(f"Framingham prepared: {len(df)} records")
    return df
```

```python
def prepare_causal_dataset(df: pd.DataFrame, dataset_name: str = "NHANES") -> pd.DataFrame:
    """Prepare a dataset for causal inference analysis.

    Creates treatment indicators, encodes categoricals, handles missing data.
    """
    df = df.copy()

    # Age filter
    if "AGE" in df.columns:
        df = df[(df["AGE"] >= AGE_MIN) & (df["AGE"] <= AGE_MAX)]

    # Create binary treatment indicators
    if "FPG" in df.columns:
        df["hyperglycemia"] = (df["FPG"] >= FPG_THRESHOLD).astype(int)

    if "SBP" in df.columns:
        htn_mask = df["SBP"] >= SBP_THRESHOLD
        if "DBP" in df.columns:
            htn_mask = htn_mask | (df["DBP"] >= DBP_THRESHOLD)
        if "ON_BP_MEDS" in df.columns:
            htn_mask = htn_mask | (df["ON_BP_MEDS"] == 1)
        df["HTN"] = htn_mask.astype(int)

    # Encode race/ethnicity as numeric (for PSM)
    if "RACE_ETH" in df.columns:
        race_dummies = pd.get_dummies(df["RACE_ETH"], prefix="race", drop_first=True)
        df = pd.concat([df, race_dummies], axis=1)
        df["RACE_ETH_encoded"] = df["RACE_ETH"].astype("category").cat.codes

    # Sex as numeric (1=Male, 0=Female for regression)
    if "SEX" in df.columns:
        df["SEX_binary"] = (df["SEX"] == 1).astype(int)

    # Smoking indicator -- use CURRENT_SMOKER if available, else keep existing SMOKING
    if "SMOKING" not in df.columns:
        if "CURRENT_SMOKER" in df.columns:
            df["SMOKING"] = df["CURRENT_SMOKER"].fillna(0).astype(int)
        else:
            logger.warning("No smoking variable available -- defaulting to 0")
            df["SMOKING"] = 0

    # Physical activity indicator
    if "PHYSICAL_ACTIVITY" not in df.columns:
        df["PHYSICAL_ACTIVITY"] = 0

    # Alcohol indicator
    if "ALCOHOL" not in df.columns:
        df["ALCOHOL"] = 0

    # Log-transform skewed variables
    if "HOMA_IR" in df.columns:
        df["HOMA_IR_log"] = np.log1p(df["HOMA_IR"].clip(lower=0))
    if "CRP" in df.columns:
        df["CRP_log"] = np.log1p(df["CRP"].clip(lower=0))

    logger.info(
        f"{dataset_name}: {len(df):,} records, "
        f"hyperglycemia={df.get('hyperglycemia', pd.Series()).sum():,}, "
        f"HTN={df.get('HTN', pd.Series()).sum():,}"
    )
    return df


def get_complete_cases(
    df: pd.DataFrame,
    required_vars: list,
) -> pd.DataFrame:
    """Return rows with no missing values in required variables."""
    available = [v for v in required_vars if v in df.columns]
    missing_vars = [v for v in required_vars if v not in df.columns]
    if missing_vars:
        logger.warning(f"Variables not in dataset: {missing_vars}")

    mask = df[available].notna().all(axis=1)
    result = df[mask].copy()
    logger.info(f"Complete cases: {len(result):,} / {len(df):,} "
                f"(required: {len(available)} vars)")
    return result
```

File: src/causal_models.py

Lines: 1016

```python
"""Causal inference models for bidirectional glucose <-> BP analysis.

Implements:
1. Structural Equation Modeling (SEM) with latent variables
2. Propensity Score Matching (PSM)
3. Inverse Probability Weighting (IPW)
4. Doubly Robust / Augmented IPW (AIPW) estimation
5. DAG conditional independence testing
6. E-value sensitivity analysis
7. Rosenbaum bounds
"""

import logging
from dataclasses import dataclass
from typing import Dict, List, Optional, Tuple

import numpy as np
import pandas as pd
from scipy import stats
import statsmodels.api as sm
from sklearn.linear_model import LogisticRegression
from sklearn.neighbors import NearestNeighbors
from sklearn.preprocessing import StandardScaler

logger = logging.getLogger(__name__)


# ============================================================
# Data classes for results
# ============================================================

@dataclass
class SEMResult:
    """Container for SEM estimation results."""
    model_description: str
    path_coefficients: Dict[str, float]
    path_std_errors: Dict[str, float]
    path_p_values: Dict[str, float]
    latent_loadings: Dict[str, Dict[str, float]]
    fit_indices: Dict[str, float]
    n_obs: int
    converged: bool


@dataclass
class PSMResult:
    """Container for propensity score matching results."""
    direction: str  # "glucose_to_bp" or "bp_to_glucose"
    treatment_name: str
    outcome_name: str
    n_treated: int
    n_control: int
    n_matched: int
    att: float  # Average Treatment Effect on the Treated
    att_se: float
    att_ci_lower: float
    att_ci_upper: float
    att_p_value: float
    smd_before: Dict[str, float]
    smd_after: Dict[str, float]
    propensity_scores: Optional[np.ndarray] = None


@dataclass
class IPWResult:
    """Container for IPW estimation results."""
    direction: str
    ate: float  # Average Treatment Effect
    ate_se: float
    ate_ci_lower: float
    ate_ci_upper: float
    ate_p_value: float
    att: float  # Average Treatment Effect on Treated
    att_se: float
    weights_summary: Dict[str, float]


@dataclass
class DoublyRobustResult:
```

```
    """Container for doubly robust (AIPW) results."""
    direction: str
    ate: float
    ate_se: float
    ate_ci_lower: float
    ate_ci_upper: float
    ate_p_value: float


@dataclass
class EValueResult:
    """Container for E-value sensitivity analysis."""
    point_estimate: float
    ci_bound: float
    e_value_point: float
    e_value_ci: float
    interpretation: str


# ============================================================
# 1. Structural Equation Modeling
# ============================================================

def build_sem_specification(
    measurement_model: Dict[str, List[str]],
    structural_paths: List[Tuple[str, str]],
) -> str:
    """Build a semopy model specification string.

    Parameters
    ----------
    measurement_model : dict
        {latent_name: [indicator1, indicator2, ...]}
    structural_paths : list
        [(from_var, to_var), ...] for structural paths

    Returns
    -------
    str
        Model specification for semopy
    """
    lines = []

    # Measurement model
    for latent, indicators in measurement_model.items():
        indicators_str = " + ".join(indicators)
        lines.append(f"{latent} =~ {indicators_str}")

    # Structural model
    # Group paths by outcome variable
    from collections import defaultdict
    outcome_predictors = defaultdict(list)
    for from_var, to_var in structural_paths:
        outcome_predictors[to_var].append(from_var)

    for outcome, predictors in outcome_predictors.items():
        predictors_str = " + ".join(predictors)
        lines.append(f"{outcome} ~ {predictors_str}")

    return "\n".join(lines)


def fit_sem(
    df: pd.DataFrame,
    model_spec: str,
    standardize: bool = True,
) -> SEMResult:
    """Fit a Structural Equation Model using semopy.

    Parameters
    ----------
    df : pd.DataFrame
        Data with all observed variables
    model_spec : str
        semopy model specification
    standardize : bool
        Whether to report standardized coefficients

    Returns
    -------
    SEMResult
    """
    try:
        import semopy
```

```python

        model = semopy.Model(model_spec)

        # Standardize data for interpretability
        df_std = df.copy()
        numeric_cols = df_std.select_dtypes(include=[np.number]).columns
        scaler = StandardScaler()
        df_std[numeric_cols] = scaler.fit_transform(df_std[numeric_cols])

        result = model.fit(df_std)

        # Extract estimates
        estimates = model.inspect()

        # Parse path coefficients
        path_coefficients = {}
        path_std_errors = {}
        path_p_values = {}
        latent_loadings = {}

        for _, row in estimates.iterrows():
            op = row["op"]
            lval = row["lval"]
            rval = row["rval"]
            est = row["Estimate"]
            se = row.get("Std. Err", np.nan)
            pv = row.get("p-value", np.nan)

            if op == "~":
                # Structural path: lval ~ rval
                key = f"{rval} -> {lval}"
                path_coefficients[key] = est
                path_std_errors[key] = se
                path_p_values[key] = pv
            elif op == "=~":
                # Measurement loading: lval =~ rval
                if lval not in latent_loadings:
                    latent_loadings[lval] = {}
                latent_loadings[lval][rval] = est

        # Fit indices
        stats_result = semopy.calc_stats(model)
        fit_indices = {}
        for col in stats_result.columns:
            try:
                fit_indices[col] = float(stats_result[col].iloc[0])
            except (ValueError, TypeError):
                pass

        return SEMResult(
            model_description=model_spec,
            path_coefficients=path_coefficients,
            path_std_errors=path_std_errors,
            path_p_values=path_p_values,
            latent_loadings=latent_loadings,
            fit_indices=fit_indices,
            n_obs=len(df_std),
            converged=True,
        )

    except ImportError:
        logger.error(
            "semopy not installed. Install with: pip install semopy\n"
            "Falling back to path analysis via OLS."
        )
        return _sem_fallback_ols(df, model_spec)

    except Exception as e:
        logger.error(f"SEM estimation failed: {e}")
        return SEMResult(
            model_description=model_spec,
            path_coefficients={},
            path_std_errors={},
            path_p_values={},
            latent_loadings={},
            fit_indices={},
            n_obs=len(df),
            converged=False,
        )


def _sem_fallback_ols(df: pd.DataFrame, model_spec: str) -> SEMResult:
    """Fallback: estimate path coefficients via sequential OLS.

```

```
    This is a simplified approximation when semopy is not available.
    """
    path_coefficients = {}
    path_std_errors = {}
    path_p_values = {}

    # Parse structural paths from specification
    for line in model_spec.split("\n"):
        line = line.strip()
        if "~" in line and "=~" not in line:
            parts = line.split("~")
            outcome = parts[0].strip()
            predictors = [p.strip() for p in parts[1].split("+")]

            available = [p for p in predictors if p in df.columns]
            if outcome in df.columns and available:
                X = sm.add_constant(df[available].dropna())
                y = df.loc[X.index, outcome]
                mask = y.notna()
                X = X[mask]
                y = y[mask]

                try:
                    model = sm.OLS(y, X).fit()
                    for pred in available:
                        key = f"{pred} -> {outcome}"
                        path_coefficients[key] = model.params.get(pred, np.nan)
                        path_std_errors[key] = model.bse.get(pred, np.nan)
                        path_p_values[key] = model.pvalues.get(pred, np.nan)
                except Exception as e:
                    logger.warning(f"OLS fallback failed for {outcome}: {e}")

    return SEMResult(
        model_description=model_spec + "\n# (OLS fallback)",
        path_coefficients=path_coefficients,
        path_std_errors=path_std_errors,
        path_p_values=path_p_values,
        latent_loadings={},
        fit_indices={"method": "OLS_fallback"},
        n_obs=len(df),
        converged=True,
    )


def sem_multigroup(
    df: pd.DataFrame,
    model_spec: str,
    group_var: str,
) -> Dict[str, SEMResult]:
    """Fit SEM separately for each group (e.g., by sex, age group)."""
    results = {}
    for group_name, group_df in df.groupby(group_var):
        if len(group_df) < 200:
            logger.warning(f"Group '{group_name}' too small ({len(group_df)}), skipping")
            continue
        result = fit_sem(group_df, model_spec)
        results[str(group_name)] = result
    return results


# ============================================================
# 2. Propensity Score Matching
# ============================================================

def estimate_propensity_scores(
    df: pd.DataFrame,
    treatment: str,
    covariates: List[str],
    method: str = "logistic",
) -> np.ndarray:
    """Estimate propensity scores P(treatment=1 | covariates).

    Parameters
    ----------
    df : pd.DataFrame
    treatment : str
        Binary treatment variable name
    covariates : list
        List of covariate names
    method : str
        "logistic" for logistic regression

    Returns
    -------
```

```
    np.ndarray
        Propensity scores
    """
    available_covs = [c for c in covariates if c in df.columns]
    if not available_covs:
        raise ValueError("No covariates available for propensity score estimation")

    X = df[available_covs].values
    y = df[treatment].values

    # Handle missing values
    mask = np.all(np.isfinite(X), axis=1) & np.isfinite(y)
    X_clean = X[mask]
    y_clean = y[mask]

    # Standardize
    scaler = StandardScaler()
    X_scaled = scaler.fit_transform(X_clean)

    # Fit logistic regression
    lr = LogisticRegression(
        max_iter=1000,
        C=1.0,
        solver="lbfgs",
        random_state=42,
    )
    lr.fit(X_scaled, y_clean)

    # Predict propensity scores for all observations
    ps = np.full(len(df), np.nan)
    X_all_scaled = scaler.transform(X)
    ps[mask] = lr.predict_proba(X_all_scaled[mask])[:, 1]

    logger.info(
        f"Propensity scores estimated: mean={np.nanmean(ps):.3f}, "
        f"std={np.nanstd(ps):.3f}, range=[{np.nanmin(ps):.3f}, {np.nanmax(ps):.3f}]"
    )
    return ps


def propensity_score_matching(
    df: pd.DataFrame,
    treatment: str,
    outcome: str,
    covariates: List[str],
    caliper: float = 0.2,
    matching_ratio: int = 1,
    direction: str = "glucose_to_bp",
) -> PSMResult:
    """Perform 1:1 nearest-neighbor propensity score matching.

    Parameters
    ----------
    df : pd.DataFrame
    treatment : str
        Binary treatment variable
    outcome : str
        Outcome variable
    covariates : list
        Covariates for propensity score model
    caliper : float
        Caliper width in SD of logit propensity score
    matching_ratio : int
        Number of controls per treated unit
    direction : str
        Label for the causal direction

    Returns
    -------
    PSMResult
    """
    available_covs = [c for c in covariates if c in df.columns]

    # Complete cases
    all_vars = [treatment, outcome] + available_covs
    mask = df[all_vars].notna().all(axis=1)
    data = df[mask].copy().reset_index(drop=True)

    if len(data) < 100:
        raise ValueError(f"Too few complete cases: {len(data)}")

    # Estimate propensity scores
    ps = estimate_propensity_scores(data, treatment, available_covs)
    data["ps"] = ps
```

```
    data = data[data["ps"].notna()].reset_index(drop=True)

    # Logit transform
    denom = (1 - data["ps"]).clip(lower=1e-10)
    data["logit_ps"] = np.log(data["ps"].clip(lower=1e-10) / denom)

    # Caliper in SD of logit PS
    caliper_width = caliper * data["logit_ps"].std()

    # Split treated and control
    treated = data[data[treatment] == 1].reset_index(drop=True)
    control = data[data[treatment] == 0].reset_index(drop=True)

    logger.info(f"PSM ({direction}): {len(treated)} treated, {len(control)} control")

    # SMD before matching
    smd_before = compute_smd(treated, control, available_covs)

    # Nearest-neighbor matching with caliper
    nn = NearestNeighbors(n_neighbors=matching_ratio, metric="euclidean")
    nn.fit(control[["logit_ps"]].values)

    distances, indices = nn.kneighbors(treated[["logit_ps"]].values)

    matched_treated_idx = []
    matched_control_idx = []
    used_controls = set()

    for i in range(len(treated)):
        for j in range(matching_ratio):
            ctrl_idx = indices[i, j]
            dist = distances[i, j]

            if dist <= caliper_width and ctrl_idx not in used_controls:
                matched_treated_idx.append(i)
                matched_control_idx.append(ctrl_idx)
                used_controls.add(ctrl_idx)
                break

    matched_treated = treated.iloc[matched_treated_idx].reset_index(drop=True)
    matched_control = control.iloc[matched_control_idx].reset_index(drop=True)

    logger.info(f"Matched: {len(matched_treated)} pairs")

    # SMD after matching
    smd_after = compute_smd(matched_treated, matched_control, available_covs)

    # Estimate ATT (Average Treatment Effect on the Treated)
    outcome_diff = matched_treated[outcome].values - matched_control[outcome].values
    att = np.mean(outcome_diff)
    att_se = np.std(outcome_diff, ddof=1) / np.sqrt(len(outcome_diff))

    # Bootstrap CI
    rng = np.random.RandomState(42)
    boot_atts = []
    for _ in range(1000):
        idx = rng.choice(len(outcome_diff), size=len(outcome_diff), replace=True)
        boot_atts.append(np.mean(outcome_diff[idx]))
    boot_atts = np.array(boot_atts)
    ci_lower = np.percentile(boot_atts, 2.5)
    ci_upper = np.percentile(boot_atts, 97.5)

    # P-value (two-sided t-test)
    t_stat = att / att_se if att_se > 0 else 0
    p_value = 2 * (1 - stats.t.cdf(abs(t_stat), df=len(outcome_diff) - 1))

    return PSMResult(
        direction=direction,
        treatment_name=treatment,
        outcome_name=outcome,
        n_treated=len(treated),
        n_control=len(control),
        n_matched=len(matched_treated),
        att=att,
        att_se=att_se,
        att_ci_lower=ci_lower,
        att_ci_upper=ci_upper,
        att_p_value=p_value,
        smd_before=smd_before,
        smd_after=smd_after,
        propensity_scores=data["ps"].values,
    )
```

```python
def compute_smd(
    treated: pd.DataFrame,
    control: pd.DataFrame,
    covariates: List[str],
) -> Dict[str, float]:
    """Compute Standardized Mean Differences for covariate balance.

    SMD = (mean_treated - mean_control) / sqrt((var_treated + var_control) / 2)
    """
    smd = {}
    for var in covariates:
        if var not in treated.columns or var not in control.columns:
            continue
        t_vals = treated[var].dropna()
        c_vals = control[var].dropna()

        if len(t_vals) < 2 or len(c_vals) < 2:
            smd[var] = np.nan
            continue

        mean_diff = t_vals.mean() - c_vals.mean()
        pooled_var = (t_vals.var() + c_vals.var()) / 2

        if pooled_var > 0:
            smd[var] = abs(mean_diff) / np.sqrt(pooled_var)
        else:
            smd[var] = 0.0

    return smd


# ============================================================
# 3. Inverse Probability Weighting (IPW)
# ============================================================

def inverse_probability_weighting(
    df: pd.DataFrame,
    treatment: str,
    outcome: str,
    covariates: List[str],
    trim_lower: float = 1,
    trim_upper: float = 99,
    direction: str = "glucose_to_bp",
) -> IPWResult:
    """Estimate ATE and ATT using stabilized IPW.

    Parameters
    ----------
    df : pd.DataFrame
    treatment : str
        Binary treatment variable
    outcome : str
        Outcome variable
    covariates : list
    trim_lower, trim_upper : float
        Percentiles for weight trimming
    direction : str
        Label for the direction

    Returns
    -------
    IPWResult
    """
    available_covs = [c for c in covariates if c in df.columns]
    all_vars = [treatment, outcome] + available_covs
    mask = df[all_vars].notna().all(axis=1)
    data = df[mask].copy()

    # Estimate propensity scores
    ps = estimate_propensity_scores(data, treatment, available_covs)
    data["ps"] = ps
    data = data[data["ps"].notna()].copy()

    # Trim extreme propensity scores
    ps_lower = np.percentile(data["ps"], trim_lower)
    ps_upper = np.percentile(data["ps"], trim_upper)
    data["ps_trimmed"] = data["ps"].clip(lower=ps_lower, upper=ps_upper)

    T = data[treatment].values
    Y = data[outcome].values
    ps_t = data["ps_trimmed"].values
    p_treat = T.mean()  # marginal probability of treatment

    # Stabilized ATE weights
```

```
    # Treated: p_treat / ps
    # Control: (1 - p_treat) / (1 - ps)
    w_ate = np.where(
        T == 1,
        p_treat / ps_t,
        (1 - p_treat) / (1 - ps_t),
    )

    # ATT weights
    # Treated: 1
    # Control: ps / (1 - ps)
    w_att = np.where(
        T == 1,
        1.0,
        ps_t / (1 - ps_t),
    )

    # ATE estimate
    ate_num = np.sum(w_ate * T * Y) / np.sum(w_ate * T) - \
              np.sum(w_ate * (1 - T) * Y) / np.sum(w_ate * (1 - T))
    ate = ate_num

    # ATT estimate
    att = (np.sum(w_att * T * Y) / np.sum(w_att * T) -
           np.sum(w_att * (1 - T) * Y) / np.sum(w_att * (1 - T)))

    # Bootstrap SE and CI
    rng = np.random.RandomState(42)
    boot_ates = []
    boot_atts = []
    n = len(data)

    for _ in range(1000):
        idx = rng.choice(n, size=n, replace=True)
        T_b, Y_b, ps_b = T[idx], Y[idx], ps_t[idx]
        p_b = T_b.mean()

        w_ate_b = np.where(T_b == 1, p_b / ps_b, (1 - p_b) / (1 - ps_b))
        w_att_b = np.where(T_b == 1, 1.0, ps_b / (1 - ps_b))

        sum_wt = np.sum(w_ate_b * T_b)
        sum_wc = np.sum(w_ate_b * (1 - T_b))
        if sum_wt > 0 and sum_wc > 0:
            ate_b = np.sum(w_ate_b * T_b * Y_b) / sum_wt - \
                    np.sum(w_ate_b * (1 - T_b) * Y_b) / sum_wc
            boot_ates.append(ate_b)

        sum_att_t = np.sum(w_att_b * T_b)
        sum_att_c = np.sum(w_att_b * (1 - T_b))
        if sum_att_t > 0 and sum_att_c > 0:
            att_b = np.sum(w_att_b * T_b * Y_b) / sum_att_t - \
                    np.sum(w_att_b * (1 - T_b) * Y_b) / sum_att_c
            boot_atts.append(att_b)

    boot_ates = np.array(boot_ates)
    ate_se = np.std(boot_ates)
    ate_ci_lower = np.percentile(boot_ates, 2.5)
    ate_ci_upper = np.percentile(boot_ates, 97.5)
    ate_p = 2 * (1 - stats.norm.cdf(abs(ate / ate_se))) if ate_se > 0 else 1.0

    att_se = np.std(boot_atts) if boot_atts else np.nan

    return IPWResult(
        direction=direction,
        ate=ate,
        ate_se=ate_se,
        ate_ci_lower=ate_ci_lower,
        ate_ci_upper=ate_ci_upper,
        ate_p_value=ate_p,
        att=att,
        att_se=att_se,
        weights_summary={
            "mean": float(np.mean(w_ate)),
            "std": float(np.std(w_ate)),
            "min": float(np.min(w_ate)),
            "max": float(np.max(w_ate)),
            "p1": float(np.percentile(w_ate, 1)),
            "p99": float(np.percentile(w_ate, 99)),
        },
    )


# ============================================================
# 4. Doubly Robust / AIPW Estimation
```

```
# ============================================================

def doubly_robust_estimator(
    df: pd.DataFrame,
    treatment: str,
    outcome: str,
    covariates: List[str],
    direction: str = "glucose_to_bp",
) -> DoublyRobustResult:
    """Augmented Inverse Probability Weighting (AIPW) estimator.

    Combines an outcome model with IPW for double robustness:
    if either the propensity score model or the outcome model
    is correctly specified, the estimator is consistent.
    """
    available_covs = [c for c in covariates if c in df.columns]
    all_vars = [treatment, outcome] + available_covs
    mask = df[all_vars].notna().all(axis=1)
    data = df[mask].copy().reset_index(drop=True)

    T = data[treatment].values
    Y = data[outcome].values

    # Propensity score model
    ps = estimate_propensity_scores(data, treatment, available_covs)
    ps = np.clip(ps, 0.01, 0.99)  # trim for stability

    # Outcome model: E[Y | X, T]
    X_covs = data[available_covs].values
    scaler = StandardScaler()
    X_scaled = scaler.fit_transform(X_covs)

    # Fit outcome model for treated
    mask_t = T == 1
    X_t = np.column_stack([X_scaled[mask_t], np.ones(mask_t.sum())])
    reg_t = sm.OLS(Y[mask_t], X_t).fit()

    # Fit outcome model for control
    mask_c = T == 0
    X_c = np.column_stack([X_scaled[mask_c], np.ones(mask_c.sum())])
    reg_c = sm.OLS(Y[mask_c], X_c).fit()

    # Predict potential outcomes for all observations
    X_all = np.column_stack([X_scaled, np.ones(len(data))])
    mu1 = reg_t.predict(X_all)  # E[Y(1) | X]
    mu0 = reg_c.predict(X_all)  # E[Y(0) | X]

    # AIPW estimator
    n = len(data)
    aipw_1 = mu1 + T * (Y - mu1) / ps
    aipw_0 = mu0 + (1 - T) * (Y - mu0) / (1 - ps)
    ate_individual = aipw_1 - aipw_0
    ate = np.mean(ate_individual)

    # Variance via influence function
    ate_se = np.std(ate_individual) / np.sqrt(n)

    ci_lower = ate - 1.96 * ate_se
    ci_upper = ate + 1.96 * ate_se
    p_value = 2 * (1 - stats.norm.cdf(abs(ate / ate_se))) if ate_se > 0 else 1.0

    return DoublyRobustResult(
        direction=direction,
        ate=ate,
        ate_se=ate_se,
        ate_ci_lower=ci_lower,
        ate_ci_upper=ci_upper,
        ate_p_value=p_value,
    )


# ============================================================
# 5. DAG Conditional Independence Testing
# ============================================================

def test_conditional_independence(
    df: pd.DataFrame,
    var_x: str,
    var_y: str,
    conditioning_set: List[str],
    method: str = "partial_correlation",
) -> Dict:
    """Test conditional independence: X ? Y | Z.

```

```
    Used to test DAG-implied conditional independencies.
    """
    all_vars = [var_x, var_y] + conditioning_set
    available = [v for v in all_vars if v in df.columns]
    if len(available) < len(all_vars):
        missing = set(all_vars) - set(available)
        return {"test": method, "p_value": np.nan, "missing_vars": list(missing)}

    data = df[available].dropna()
    if len(data) < 30:
        return {"test": method, "p_value": np.nan, "error": "insufficient data"}

    if method == "partial_correlation":
        return _partial_correlation_test(data, var_x, var_y, conditioning_set)
    else:
        raise ValueError(f"Unknown method: {method}")


def _partial_correlation_test(
    data: pd.DataFrame,
    var_x: str,
    var_y: str,
    conditioning_set: List[str],
) -> Dict:
    """Test conditional independence via partial correlation.

    Regresses X and Y on Z, then tests correlation of residuals.
    """
    if not conditioning_set:
        # Simple correlation test
        r, p = stats.pearsonr(data[var_x], data[var_y])
        return {
            "test": "pearson_correlation",
            "statistic": r,
            "p_value": p,
            "n": len(data),
            "independent": p > 0.05,
        }

    # Partial correlation via residuals
    Z = sm.add_constant(data[conditioning_set])

    # Regress X on Z
    model_x = sm.OLS(data[var_x], Z).fit()
    resid_x = model_x.resid

    # Regress Y on Z
    model_y = sm.OLS(data[var_y], Z).fit()
    resid_y = model_y.resid

    # Correlation of residuals
    r, p = stats.pearsonr(resid_x, resid_y)

    return {
        "test": "partial_correlation",
        "var_x": var_x,
        "var_y": var_y,
        "conditioning_set": conditioning_set,
        "partial_r": r,
        "p_value": p,
        "n": len(data),
        "independent": p > 0.05,
    }


def test_dag_implications(
    df: pd.DataFrame,
    dag_edges: List[Tuple[str, str]],
    nodes: List[str],
) -> pd.DataFrame:
    """Test all testable conditional independencies implied by a DAG.

    For each pair of non-adjacent nodes, tests whether they are
    conditionally independent given their mutual parents.
    """
    # Build adjacency set
    adjacent = set()
    parents = {n: [] for n in nodes}
    for u, v in dag_edges:
        adjacent.add((u, v))
        adjacent.add((v, u))
        parents[v].append(u)

    results = []
```

```python
    tested_pairs = set()

    for x in nodes:
        for y in nodes:
            if x >= y:
                continue
            if (x, y) in tested_pairs:
                continue
            if (x, y) in adjacent or (y, x) in adjacent:
                continue

            tested_pairs.add((x, y))

            # Conditioning set: parents of both X and Y
            cond_set = list(set(parents.get(x, []) + parents.get(y, [])) - {x, y})

            result = test_conditional_independence(df, x, y, cond_set)
            result["var_x"] = x
            result["var_y"] = y
            result["conditioning_set"] = ", ".join(cond_set) if cond_set else "(none)"
            results.append(result)

    return pd.DataFrame(results)


# ============================================================
# 6. E-value Sensitivity Analysis
# ============================================================

def compute_evalue(
    point_estimate: float,
    ci_bound: float,
    outcome_type: str = "continuous",
    outcome_sd: float = 1.0,
) -> EValueResult:
    """Compute the E-value for sensitivity to unmeasured confounding.

    The E-value is the minimum strength of association (on the RR scale)
    that an unmeasured confounder would need to have with both the
    treatment and outcome to fully explain the observed effect.

    Parameters
    ----------
    point_estimate : float
        Point estimate of the effect (mean difference for continuous,
        risk ratio for binary)
    ci_bound : float
        Confidence interval bound closest to null (for continuous: the
        lower bound if effect > 0)
    outcome_type : str
        "continuous" or "binary"
    outcome_sd : float
        SD of outcome (for continuous outcomes, to convert to RR scale)
    """
    if outcome_type == "continuous":
        # Convert mean difference to approximate RR
        # Using VanderWeele's approximation: RR ? exp(0.91 * d / SD)
        rr_point = np.exp(0.91 * point_estimate / outcome_sd)
        rr_ci = np.exp(0.91 * ci_bound / outcome_sd)
    else:
        rr_point = point_estimate
        rr_ci = ci_bound

    # E-value formula: RR + sqrt(RR * (RR - 1))
    def _evalue(rr):
        if rr < 1:
            rr = 1.0 / rr  # flip if protective
        return rr + np.sqrt(rr * (rr - 1))

    e_point = _evalue(rr_point)

    if (rr_ci >= 1.0 and point_estimate > 0) or (rr_ci <= 1.0 and point_estimate < 0):
        # CI does not cross null
        e_ci = _evalue(rr_ci)
    else:
        # CI crosses null -- E-value for CI is 1.0
        e_ci = 1.0

    # Interpretation
    if e_point > 3.0:
        strength = "very strong"
    elif e_point > 2.0:
        strength = "strong"
    elif e_point > 1.5:
```

```
        strength = "moderate"
    else:
        strength = "weak"

    interpretation = (
        f"An unmeasured confounder would need to be associated with both "
        f"the treatment and the outcome by a risk ratio of at least "
        f"{e_point:.2f} (point estimate) or {e_ci:.2f} (confidence interval "
        f"limit) to fully explain the observed effect. This represents "
        f"{strength} evidence against confounding as the sole explanation."
    )

    return EValueResult(
        point_estimate=rr_point,
        ci_bound=rr_ci,
        e_value_point=e_point,
        e_value_ci=e_ci,
        interpretation=interpretation,
    )


# ============================================================
# 7. Rosenbaum Bounds
# ============================================================

def rosenbaum_bounds(
    matched_treated: np.ndarray,
    matched_control: np.ndarray,
    gamma_range: Optional[List[float]] = None,
) -> pd.DataFrame:
    """Sensitivity analysis using Rosenbaum bounds.

    Tests how much hidden bias (?) could alter the conclusion
    of the matched pair analysis.

    Parameters
    ----------
    matched_treated : np.ndarray
        Outcomes for treated units in matched pairs
    matched_control : np.ndarray
        Outcomes for control units in matched pairs
    gamma_range : list
        Values of ? (sensitivity parameter) to evaluate

    Returns
    -------
    pd.DataFrame
        Columns: gamma, p_upper, p_lower, significant
    """
    if gamma_range is None:
        gamma_range = [1.0, 1.1, 1.2, 1.3, 1.4, 1.5, 1.6, 1.8, 2.0, 2.5, 3.0]

    diffs = matched_treated - matched_control
    n = len(diffs)
    ranks = stats.rankdata(np.abs(diffs))

    results = []
    for gamma in gamma_range:
        # Under ?-sensitivity model:
        # Upper bound: p+ = ? / (1 + ?)
        # Lower bound: p+ = 1 / (1 + ?)

        # Wilcoxon signed-rank test statistic
        T_plus = np.sum(ranks[diffs > 0])

        # Expected value and variance under ?
        p_upper = gamma / (1 + gamma)
        p_lower = 1.0 / (1 + gamma)

        # E[T+] and Var[T+] under the sensitivity model
        E_upper = np.sum(ranks * p_upper)
        V_upper = np.sum(ranks**2 * p_upper * (1 - p_upper))

        E_lower = np.sum(ranks * p_lower)
        V_lower = np.sum(ranks**2 * p_lower * (1 - p_lower))

        # Z-scores
        if V_upper > 0:
            z_upper = (T_plus - E_upper) / np.sqrt(V_upper)
            p_val_upper = 1 - stats.norm.cdf(z_upper)
        else:
            p_val_upper = 1.0

        if V_lower > 0:
```

```
            z_lower = (T_plus - E_lower) / np.sqrt(V_lower)
            p_val_lower = 1 - stats.norm.cdf(z_lower)
        else:
            p_val_lower = 1.0

        results.append({
            "gamma": gamma,
            "p_upper": p_val_upper,
            "p_lower": p_val_lower,
            "significant_at_005": p_val_upper < 0.05,
        })

    return pd.DataFrame(results)
```

File: src/evaluation.py

Lines: 444

```python
"""Evaluation and table generation for the causal inference article.

Generates all 8 tables and summary statistics.
"""

import logging
from pathlib import Path
from typing import Dict, List, Optional

import numpy as np
import pandas as pd

from .config import (
    TABLES_DIR, FPG_THRESHOLD, SBP_THRESHOLD, DBP_THRESHOLD,
    SEM_FIT_THRESHOLDS,
)
from .causal_models import (
    SEMResult, PSMResult, IPWResult, DoublyRobustResult, EValueResult,
)

logger = logging.getLogger(__name__)


def save_table(df: pd.DataFrame, name: str, output_dir: Path = None):
    """Save table as CSV and LaTeX."""
    if output_dir is None:
        output_dir = TABLES_DIR
    output_dir.mkdir(parents=True, exist_ok=True)

    df.to_csv(output_dir / f"{name}.csv", index=False)
    try:
        df.to_latex(output_dir / f"{name}.tex", index=False, escape=True)
    except Exception:
        pass
    logger.info(f"Saved: {name}.csv")


def generate_table1_baseline(
    df: pd.DataFrame,
    output_dir: Path = None,
) -> pd.DataFrame:
    """Table 1: Baseline characteristics by hyperglycemia and hypertension status.

    Four groups: (1) Neither, (2) Hyperglycemia only, (3) HTN only, (4) Both
    """
    df = df.copy()

    # Define groups
    has_hyper = df.get("hyperglycemia", pd.Series(dtype=int)) == 1
    has_htn = df.get("HTN", pd.Series(dtype=int)) == 1

    groups = {
        "Neither": ~has_hyper & ~has_htn,
        "Hyperglycemia Only": has_hyper & ~has_htn,
        "Hypertension Only": ~has_hyper & has_htn,
        "Both": has_hyper & has_htn,
    }

    continuous_vars = [
        ("AGE", "Age (years)"),
        ("BMI", "BMI (kg/m^2)"),
        ("WAIST", "Waist circumference (cm)"),
        ("FPG", "Fasting plasma glucose (mg/dL)"),
        ("HBA1C", "HbA1c (%)"),
        ("HOMA_IR", "HOMA-IR"),
        ("SBP", "Systolic BP (mmHg)"),
        ("DBP", "Diastolic BP (mmHg)"),
        ("TOTAL_CHOL", "Total cholesterol (mg/dL)"),
        ("HDL_CHOL", "HDL cholesterol (mg/dL)"),
        ("CRP", "C-reactive protein (mg/dL)"),
    ]

    rows = []
    rows.append({
        "Variable": "n",
        **{name: f"{mask.sum():,}" for name, mask in groups.items()},
    })

    for var, label in continuous_vars:
```

```
        if var not in df.columns:
            continue
        row = {"Variable": label}
        for name, mask in groups.items():
            vals = df.loc[mask, var].dropna()
            if len(vals) > 0:
                row[name] = f"{vals.mean():.1f} +/- {vals.std():.1f}"
            else:
                row[name] = "--"
        rows.append(row)

    # Binary variables
    binary_vars = [
        ("SEX", 2, "Female sex, n (%)"),
        ("ON_BP_MEDS", 1, "On BP medication, n (%)"),
        ("SMOKING", 1, "Current smoker, n (%)"),
    ]

    for var, val, label in binary_vars:
        if var not in df.columns:
            continue
        row = {"Variable": label}
        for name, mask in groups.items():
            total = mask.sum()
            count = (df.loc[mask, var] == val).sum()
            pct = 100 * count / total if total > 0 else 0
            row[name] = f"{count:,} ({pct:.1f}%)"
        rows.append(row)

    result = pd.DataFrame(rows)
    save_table(result, "table1_baseline", output_dir)
    return result


def generate_table2_dag_tests(
    dag_test_results: pd.DataFrame,
    output_dir: Path = None,
) -> pd.DataFrame:
    """Table 2: DAG conditional independence test results."""
    cols = ["var_x", "var_y", "conditioning_set", "partial_r", "p_value", "independent"]
    available_cols = [c for c in cols if c in dag_test_results.columns]

    df = dag_test_results[available_cols].copy()
    df = df.rename(columns={
        "var_x": "Variable X",
        "var_y": "Variable Y",
        "conditioning_set": "Conditioning Set",
        "partial_r": "Partial r",
        "p_value": "p-value",
        "independent": "Independent (p > 0.05)",
    })

    if "p-value" in df.columns:
        df["p-value"] = df["p-value"].apply(
            lambda x: f"{x:.4f}" if not np.isnan(x) else "--"
        )
    if "Partial r" in df.columns:
        df["Partial r"] = df["Partial r"].apply(
            lambda x: f"{x:.3f}" if not np.isnan(x) else "--"
        )

    save_table(df, "table2_dag_tests", output_dir)
    return df


def generate_table3_sem(
    sem_result: SEMResult,
    output_dir: Path = None,
) -> pd.DataFrame:
    """Table 3: SEM standardized path coefficients with fit indices."""
    rows = []

    # Path coefficients
    for path, coeff in sem_result.path_coefficients.items():
        se = sem_result.path_std_errors.get(path, np.nan)
        pv = sem_result.path_p_values.get(path, np.nan)

        # Safe nan check for mixed types
        def _is_nan(v):
            try:
                return v is None or (isinstance(v, (float, np.floating)) and np.isnan(v))
            except (TypeError, ValueError):
                return False

```

```
        sig = ""
        if not _is_nan(pv):
            try:
                pv_f = float(pv)
                if pv_f < 0.001:
                    sig = "***"
                elif pv_f < 0.01:
                    sig = "**"
                elif pv_f < 0.05:
                    sig = "*"
            except (TypeError, ValueError):
                pass

        try:
            coeff_str = f"{float(coeff):.4f}"
        except (TypeError, ValueError):
            coeff_str = str(coeff)

        try:
            se_str = f"{float(se):.4f}" if not _is_nan(se) else "--"
        except (TypeError, ValueError):
            se_str = str(se)

        try:
            pv_str = f"{float(pv):.4f}" if not _is_nan(pv) else "--"
        except (TypeError, ValueError):
            pv_str = str(pv)

        rows.append({
            "Path": path,
            "Standardized beta": coeff_str,
            "SE": se_str,
            "p-value": pv_str,
            "Significance": sig,
        })

    # Add fit indices as footer rows
    rows.append({"Path": "--- Fit Indices ---", "Standardized beta": "", "SE": "", "p-value": "", "Significance": ""})

    for key, value in sem_result.fit_indices.items():
        try:
            val_f = float(value)
        except (ValueError, TypeError):
            continue

        if key in SEM_FIT_THRESHOLDS:
            threshold = SEM_FIT_THRESHOLDS[key]
            acceptable = "?" if (
                (key in ["RMSEA", "SRMR"] and val_f < threshold) or
                (key in ["CFI", "TLI"] and val_f > threshold)
            ) else "?"
        else:
            acceptable = ""

        rows.append({
            "Path": key,
            "Standardized beta": f"{val_f:.4f}",
            "SE": f"(threshold: {SEM_FIT_THRESHOLDS.get(key, '--')})",
            "p-value": acceptable,
            "Significance": "",
        })

    rows.append({
        "Path": "n",
        "Standardized beta": f"{sem_result.n_obs:,}",
        "SE": "", "p-value": "", "Significance": "",
    })

    result = pd.DataFrame(rows)
    save_table(result, "table3_sem_results", output_dir)
    return result


def generate_table4_psm_glucose_bp(
    psm_result: PSMResult,
    output_dir: Path = None,
) -> pd.DataFrame:
    """Table 4: PSM results for glucose -> BP direction."""
    rows = [
        {"Metric": "Direction", "Value": "Hyperglycemia -> Blood Pressure"},
        {"Metric": "Treatment", "Value": f"FPG >= {FPG_THRESHOLD} mg/dL"},
        {"Metric": "Outcome", "Value": psm_result.outcome_name},
        {"Metric": "N (treated)", "Value": f"{psm_result.n_treated:,}"},
        {"Metric": "N (control)", "Value": f"{psm_result.n_control:,}"},
```

```
        {"Metric": "N (matched pairs)", "Value": f"{psm_result.n_matched:,}"},
        {"Metric": "ATT (mmHg)", "Value": f"{psm_result.att:.2f}"},
        {"Metric": "SE", "Value": f"{psm_result.att_se:.2f}"},
        {"Metric": "95% CI", "Value": f"({psm_result.att_ci_lower:.2f}, {psm_result.att_ci_upper:.2f})"},
        {"Metric": "p-value", "Value": f"{psm_result.att_p_value:.4f}"},
    ]

    # SMD summary
    n_balanced = sum(1 for v in psm_result.smd_after.values() if v < 0.1)
    n_total = len(psm_result.smd_after)
    rows.append({
        "Metric": "Covariates balanced (SMD < 0.1)",
        "Value": f"{n_balanced}/{n_total}",
    })

    result = pd.DataFrame(rows)
    save_table(result, "table4_psm_glucose_bp", output_dir)
    return result


def generate_table5_ipw_dr(
    ipw_g2b: IPWResult,
    ipw_b2g: IPWResult,
    dr_g2b: DoublyRobustResult,
    dr_b2g: DoublyRobustResult,
    output_dir: Path = None,
) -> pd.DataFrame:
    """Table 5: IPW and doubly robust estimates for both directions."""
    rows = []

    for method, g2b, b2g in [
        ("IPW (ATE)", ipw_g2b, ipw_b2g),
        ("Doubly Robust (AIPW)", dr_g2b, dr_b2g),
    ]:
        # Glucose -> BP
        rows.append({
            "Method": method,
            "Direction": "Glucose -> BP",
            "Estimate": f"{g2b.ate:.2f}" if hasattr(g2b, 'ate') else "--",
            "SE": f"{g2b.ate_se:.2f}" if hasattr(g2b, 'ate_se') else "--",
            "95% CI": f"({g2b.ate_ci_lower:.2f}, {g2b.ate_ci_upper:.2f})",
            "p-value": f"{g2b.ate_p_value:.4f}" if hasattr(g2b, 'ate_p_value') else "--",
        })

        # BP -> Glucose
        rows.append({
            "Method": method,
            "Direction": "BP -> Glucose",
            "Estimate": f"{b2g.ate:.2f}" if hasattr(b2g, 'ate') else "--",
            "SE": f"{b2g.ate_se:.2f}" if hasattr(b2g, 'ate_se') else "--",
            "95% CI": f"({b2g.ate_ci_lower:.2f}, {b2g.ate_ci_upper:.2f})",
            "p-value": f"{b2g.ate_p_value:.4f}" if hasattr(b2g, 'ate_p_value') else "--",
        })

    result = pd.DataFrame(rows)
    save_table(result, "table5_ipw_doubly_robust", output_dir)
    return result


def generate_table6_psm_bp_glucose(
    psm_result: PSMResult,
    output_dir: Path = None,
) -> pd.DataFrame:
    """Table 6: PSM results for BP -> glucose direction."""
    rows = [
        {"Metric": "Direction", "Value": "Hypertension -> Blood Glucose"},
        {"Metric": "Treatment", "Value": f"SBP >= {SBP_THRESHOLD} or DBP >= {DBP_THRESHOLD} or on BP meds"},
        {"Metric": "Outcome", "Value": psm_result.outcome_name},
        {"Metric": "N (treated)", "Value": f"{psm_result.n_treated:,}"},
        {"Metric": "N (control)", "Value": f"{psm_result.n_control:,}"},
        {"Metric": "N (matched pairs)", "Value": f"{psm_result.n_matched:,}"},
        {"Metric": "ATT (mg/dL)", "Value": f"{psm_result.att:.2f}"},
        {"Metric": "SE", "Value": f"{psm_result.att_se:.2f}"},
        {"Metric": "95% CI", "Value": f"({psm_result.att_ci_lower:.2f}, {psm_result.att_ci_upper:.2f})"},
        {"Metric": "p-value", "Value": f"{psm_result.att_p_value:.4f}"},
    ]

    n_balanced = sum(1 for v in psm_result.smd_after.values() if v < 0.1)
    n_total = len(psm_result.smd_after)
    rows.append({
        "Metric": "Covariates balanced (SMD < 0.1)",
        "Value": f"{n_balanced}/{n_total}",
    })

```

```
    result = pd.DataFrame(rows)
    save_table(result, "table6_psm_bp_glucose", output_dir)
    return result


def generate_table7_evalue(
    evalue_g2b: EValueResult,
    evalue_b2g: EValueResult,
    rosenbaum_g2b: pd.DataFrame,
    rosenbaum_b2g: pd.DataFrame,
    output_dir: Path = None,
) -> pd.DataFrame:
    """Table 7: E-values and Rosenbaum ? for all estimates."""
    rows = [
        {
            "Direction": "Glucose -> BP",
            "Observed RR": f"{evalue_g2b.point_estimate:.2f}",
            "E-value (point)": f"{evalue_g2b.e_value_point:.2f}",
            "E-value (CI)": f"{evalue_g2b.e_value_ci:.2f}",
            "Max ? (p < 0.05)": _max_gamma(rosenbaum_g2b),
        },
        {
            "Direction": "BP -> Glucose",
            "Observed RR": f"{evalue_b2g.point_estimate:.2f}",
            "E-value (point)": f"{evalue_b2g.e_value_point:.2f}",
            "E-value (CI)": f"{evalue_b2g.e_value_ci:.2f}",
            "Max ? (p < 0.05)": _max_gamma(rosenbaum_b2g),
        },
    ]

    result = pd.DataFrame(rows)
    save_table(result, "table7_evalue", output_dir)
    return result


def _max_gamma(bounds_df: pd.DataFrame) -> str:
    """Find maximum ? where result remains significant."""
    if bounds_df is None or bounds_df.empty:
        return "--"
    sig = bounds_df[bounds_df["significant_at_005"]]
    if sig.empty:
        return "< 1.0"
    return f"{sig['gamma'].max():.1f}"


def generate_table8_framingham(
    nhanes_sem: SEMResult,
    framingham_sem: SEMResult,
    output_dir: Path = None,
) -> pd.DataFrame:
    """Table 8: SEM comparison NHANES vs Framingham."""
    all_paths = sorted(
        set(nhanes_sem.path_coefficients.keys()) |
        set(framingham_sem.path_coefficients.keys())
    )

    def _safe_float(v):
        try:
            return float(v)
        except (TypeError, ValueError):
            return np.nan

    def _fmt(v, fmt=".4f"):
        f = _safe_float(v)
        return f"{f:{fmt}}" if not np.isnan(f) else "--"

    rows = []
    for path in all_paths:
        n_coeff = _safe_float(nhanes_sem.path_coefficients.get(path, np.nan))
        n_pval = _safe_float(nhanes_sem.path_p_values.get(path, np.nan))
        f_coeff = _safe_float(framingham_sem.path_coefficients.get(path, np.nan))
        f_pval = _safe_float(framingham_sem.path_p_values.get(path, np.nan))

        consistent = "--"
        if not np.isnan(n_coeff) and not np.isnan(f_coeff):
            consistent = "?" if np.sign(n_coeff) == np.sign(f_coeff) else "?"

        rows.append({
            "Path": path,
            "NHANES beta": _fmt(n_coeff),
            "NHANES p": _fmt(n_pval),
            "Framingham beta": _fmt(f_coeff),
            "Framingham p": _fmt(f_pval),
            "Direction Consistent": consistent,
```

```
        })

    # Add fit indices comparison
    rows.append({"Path": "--- Fit Indices ---", "NHANES beta": "", "NHANES p": "",
                 "Framingham beta": "", "Framingham p": "", "Direction Consistent": ""})
    rows.append({
        "Path": "n",
        "NHANES beta": f"{nhanes_sem.n_obs:,}",
        "NHANES p": "",
        "Framingham beta": f"{framingham_sem.n_obs:,}",
        "Framingham p": "",
        "Direction Consistent": "",
    })

    for key in ["RMSEA", "CFI", "TLI", "SRMR"]:
        n_val = _safe_float(nhanes_sem.fit_indices.get(key, np.nan))
        f_val = _safe_float(framingham_sem.fit_indices.get(key, np.nan))
        rows.append({
            "Path": key,
            "NHANES beta": _fmt(n_val),
            "NHANES p": "",
            "Framingham beta": _fmt(f_val),
            "Framingham p": "",
            "Direction Consistent": "",
        })

    result = pd.DataFrame(rows)
    save_table(result, "table8_framingham", output_dir)
    return result
```

File: src/visualization.py

Lines: 647

```python
"""Visualization functions for the bidirectional causal inference article.

Generates all 8 figures:
1. DAG diagram
2. SEM path diagram
3-4. Love plots (covariate balance)
5. E-value contour plots
6. NHANES vs Framingham SEM comparison
7. Propensity score overlap
8. Rosenbaum bounds sensitivity
"""

import logging
from pathlib import Path
from typing import Dict, List, Optional, Tuple

import matplotlib as mpl
import matplotlib.pyplot as plt
import matplotlib.patches as mpatches
import numpy as np
import pandas as pd

from .config import COLORBLIND_PALETTE, FIG_DPI, FIGURES_DIR, SMD_THRESHOLD

logger = logging.getLogger(__name__)

# MDPI compliance: Unicode minus signs
mpl.rcParams["axes.unicode_minus"] = True
mpl.rcParams["font.family"] = "sans-serif"
mpl.rcParams["font.size"] = 10


def save_figure(fig, name: str, output_dir: Path = None):
    """Save figure as both PNG and PDF."""
    if output_dir is None:
        output_dir = FIGURES_DIR
    output_dir.mkdir(parents=True, exist_ok=True)

    fig.savefig(output_dir / f"{name}.png", dpi=FIG_DPI, bbox_inches="tight")
    fig.savefig(output_dir / f"{name}.pdf", bbox_inches="tight")
    logger.info(f"Saved: {name}.png and {name}.pdf")
    plt.close(fig)


def plot_dag(
    nodes: List[str],
    edges: List[Tuple[str, str]],
    highlight_edges: Optional[List[Tuple[str, str]]] = None,
    node_positions: Optional[Dict[str, Tuple[float, float]]] = None,
    output_dir: Path = None,
):
    """Plot the Directed Acyclic Graph (Figure 1).

    Parameters
    ----------
    nodes : list of str
    edges : list of (from, to)
    highlight_edges : list of (from, to) to highlight in a different color
    node_positions : dict of {node: (x, y)}
    """
    fig, ax = plt.subplots(1, 1, figsize=(10, 7))

    if node_positions is None:
        # Default layout: conceptual grouping
        node_positions = {
            # Exposures/outcomes (center)
            "FPG": (0.35, 0.5),
            "HBA1C": (0.2, 0.35),
            "SBP": (0.65, 0.5),
            "DBP": (0.8, 0.35),
            # Metabolic syndrome (top center)
            "BMI": (0.35, 0.85),
            "WAIST": (0.5, 0.95),
            "HOMA_IR": (0.5, 0.7),
            # Demographics (left)
            "AGE": (0.05, 0.7),
            "SEX": (0.05, 0.5),
            # Others (right/bottom)
            "ON_BP_MEDS": (0.85, 0.7),
```

```
            "TOTAL_CHOL": (0.75, 0.85),
            "HDL_CHOL": (0.9, 0.85),
            "CRP": (0.5, 0.15),
        }

    if highlight_edges is None:
        highlight_edges = [("FPG", "SBP"), ("FPG", "DBP"), ("HBA1C", "SBP")]

    highlight_set = set(highlight_edges)

    # Draw edges
    for u, v in edges:
        if u not in node_positions or v not in node_positions:
            continue
        x1, y1 = node_positions[u]
        x2, y2 = node_positions[v]

        is_highlight = (u, v) in highlight_set
        color = COLORBLIND_PALETTE[4] if is_highlight else "#666666"
        linewidth = 2.5 if is_highlight else 1.0
        alpha = 1.0 if is_highlight else 0.5

        ax.annotate(
            "",
            xy=(x2, y2),
            xytext=(x1, y1),
            arrowprops=dict(
                arrowstyle="-|>",
                color=color,
                lw=linewidth,
                alpha=alpha,
                connectionstyle="arc3,rad=0.1",
            ),
        )

    # Draw nodes
    node_colors = {
        "FPG": COLORBLIND_PALETTE[0],
        "HBA1C": COLORBLIND_PALETTE[0],
        "SBP": COLORBLIND_PALETTE[4],
        "DBP": COLORBLIND_PALETTE[4],
        "HOMA_IR": COLORBLIND_PALETTE[1],
        "BMI": COLORBLIND_PALETTE[1],
        "WAIST": COLORBLIND_PALETTE[1],
    }

    node_labels = {
        "FPG": "FPG",
        "HBA1C": "HbA1c",
        "SBP": "SBP",
        "DBP": "DBP",
        "BMI": "BMI",
        "WAIST": "Waist",
        "HOMA_IR": "HOMA-IR",
        "AGE": "Age",
        "SEX": "Sex",
        "ON_BP_MEDS": "BP Meds",
        "TOTAL_CHOL": "Total Chol",
        "HDL_CHOL": "HDL Chol",
        "CRP": "CRP",
    }

    for node in nodes:
        if node not in node_positions:
            continue
        x, y = node_positions[node]
        color = node_colors.get(node, "#CCCCCC")
        label = node_labels.get(node, node)

        circle = mpatches.FancyBboxPatch(
            (x - 0.05, y - 0.03), 0.10, 0.06,
            boxstyle="round,pad=0.01",
            facecolor=color,
            edgecolor="black",
            linewidth=1.5,
            alpha=0.85,
        )
        ax.add_patch(circle)
        ax.text(x, y, label, ha="center", va="center",
                fontsize=8, fontweight="bold", color="white")

    # Legend
    legend_elements = [
        mpatches.Patch(facecolor=COLORBLIND_PALETTE[0], label="Glycemic variables"),
```

```
        mpatches.Patch(facecolor=COLORBLIND_PALETTE[4], label="Blood pressure variables"),
        mpatches.Patch(facecolor=COLORBLIND_PALETTE[1], label="Metabolic variables"),
        mpatches.Patch(facecolor="#CCCCCC", label="Covariates"),
        plt.Line2D([0], [0], color=COLORBLIND_PALETTE[4], lw=2.5,
                   label="Paths of interest (glucose -> BP)"),
        plt.Line2D([0], [0], color="#666666", lw=1.0, alpha=0.5,
                   label="Confounding/adjustment paths"),
    ]
    ax.legend(handles=legend_elements, loc="lower left", fontsize=8,
              framealpha=0.9)

    ax.set_xlim(-0.05, 1.05)
    ax.set_ylim(0.0, 1.05)
    ax.set_aspect("equal")
    ax.axis("off")
    ax.set_title("Directed Acyclic Graph: Glucose-Blood Pressure Causal Structure",
                 fontsize=12, fontweight="bold", pad=15)

    save_figure(fig, "fig1_dag", output_dir)


def plot_sem_path_diagram(
    sem_result,
    output_dir: Path = None,
):
    """Plot SEM path diagram with standardized coefficients (Figure 2)."""
    fig, ax = plt.subplots(1, 1, figsize=(10, 7))

    # Position latent variables
    latent_pos = {
        "MetS": (0.5, 0.85),
        "Glycemic": (0.2, 0.45),
        "BPState": (0.8, 0.45),
    }

    # Draw latent variable ellipses
    for latent, (x, y) in latent_pos.items():
        ellipse = mpatches.Ellipse(
            (x, y), 0.22, 0.12,
            facecolor=COLORBLIND_PALETTE[5],
            edgecolor="black",
            linewidth=2,
            alpha=0.8,
        )
        ax.add_patch(ellipse)
        ax.text(x, y, latent, ha="center", va="center",
                fontsize=11, fontweight="bold")

    # Draw structural paths with coefficients
    if sem_result.converged and sem_result.path_coefficients:
        paths_to_draw = [
            ("MetS", "Glycemic"),
            ("MetS", "BPState"),
            ("Glycemic", "BPState"),
        ]

        for from_var, to_var in paths_to_draw:
            key = f"{from_var} -> {to_var}"
            coeff = sem_result.path_coefficients.get(key, None)
            p_val = sem_result.path_p_values.get(key, 1.0)

            x1, y1 = latent_pos[from_var]
            x2, y2 = latent_pos[to_var]

            is_significant = p_val < 0.05 if not np.isnan(p_val) else False
            color = COLORBLIND_PALETTE[0] if is_significant else "#999999"
            style = "solid" if is_significant else "dashed"

            ax.annotate(
                "",
                xy=(x2, y2),
                xytext=(x1, y1),
                arrowprops=dict(
                    arrowstyle="-|>",
                    color=color,
                    lw=2.5,
                    linestyle=style,
                    connectionstyle="arc3,rad=0.15",
                ),
            )

            # Label with coefficient and 95% CI
            if coeff is not None:
                mid_x = (x1 + x2) / 2
```

```
                mid_y = (y1 + y2) / 2 + 0.05
                stars = ""
                try:
                    pv_f = float(p_val)
                    stars = "***" if pv_f < 0.001 else "**" if pv_f < 0.01 else "*" if pv_f < 0.05 else ""
                except (TypeError, ValueError):
                    pass

                # Build label with CI if SE available
                se = sem_result.path_std_errors.get(key, None)
                try:
                    coeff_f = float(coeff)
                    if se is not None:
                        se_f = float(se)
                        ci_lo = coeff_f - 1.96 * se_f
                        ci_hi = coeff_f + 1.96 * se_f
                        label_text = f"\u03b2 = {coeff_f:.3f}{stars}\n95% CI: ({ci_lo:.3f}, {ci_hi:.3f})"
                    else:
                        label_text = f"\u03b2 = {coeff_f:.3f}{stars}"
                except (TypeError, ValueError):
                    label_text = f"\u03b2 = {coeff}{stars}"

                ax.text(mid_x, mid_y, label_text,
                        ha="center", va="center", fontsize=8,
                        bbox=dict(boxstyle="round,pad=0.2",
                                  facecolor="white", edgecolor=color, alpha=0.9))

    # Draw indicator variables
    indicators = {
        "MetS": ["BMI", "Waist", "HOMA-IR"],
        "Glycemic": ["FPG", "HbA1c"],
        "BPState": ["SBP", "DBP"],
    }

    for latent, inds in indicators.items():
        lx, ly = latent_pos[latent]
        n = len(inds)
        for i, ind in enumerate(inds):
            ix = lx + (i - (n - 1) / 2) * 0.12
            iy = ly - 0.22

            rect = mpatches.FancyBboxPatch(
                (ix - 0.05, iy - 0.025), 0.10, 0.05,
                boxstyle="round,pad=0.005",
                facecolor="white",
                edgecolor="black",
                linewidth=1,
            )
            ax.add_patch(rect)
            ax.text(ix, iy, ind, ha="center", va="center", fontsize=8)

            # Arrow from latent to indicator
            ax.annotate(
                "",
                xy=(ix, iy + 0.025),
                xytext=(lx, ly - 0.06),
                arrowprops=dict(arrowstyle="-|>", color="#666666", lw=1.0),
            )

    ax.set_xlim(-0.05, 1.05)
    ax.set_ylim(0.05, 1.0)
    ax.set_aspect("equal")
    ax.axis("off")
    ax.set_title("Structural Equation Model: Path Diagram with Standardized Coefficients",
                 fontsize=12, fontweight="bold", pad=15)

    # Fit indices annotation
    if sem_result.fit_indices:
        fit_text_parts = []
        for key in ["RMSEA", "CFI", "TLI", "SRMR"]:
            if key in sem_result.fit_indices:
                fit_text_parts.append(f"{key} = {sem_result.fit_indices[key]:.3f}")
        if fit_text_parts:
            fit_text = "Fit: " + ", ".join(fit_text_parts)
            ax.text(0.5, 0.02, fit_text, ha="center", fontsize=9,
                    style="italic", transform=ax.transAxes)

    save_figure(fig, "fig2_sem_path_diagram", output_dir)


def plot_love_plot(
    smd_before: Dict[str, float],
    smd_after: Dict[str, float],
    threshold: float = SMD_THRESHOLD,
```

```python
    title: str = "Covariate Balance: Before and After Matching",
    filename: str = "fig3_love_plot",
    output_dir: Path = None,
):
    """Plot a Love plot showing covariate balance (Figures 3-4).

    Shows standardized mean differences before and after propensity
    score matching, with a threshold line.
    """
    # Combine and sort by before-matching SMD
    covariates = sorted(
        set(smd_before.keys()) & set(smd_after.keys()),
        key=lambda x: abs(smd_before.get(x, 0)),
        reverse=True,
    )

    if not covariates:
        logger.warning("No covariates to plot in Love plot")
        return

    fig, ax = plt.subplots(1, 1, figsize=(8, max(4, len(covariates) * 0.35)))

    y_pos = np.arange(len(covariates))
    before_vals = [abs(smd_before.get(c, 0)) for c in covariates]
    after_vals = [abs(smd_after.get(c, 0)) for c in covariates]

    # Clean labels
    label_map = {
        "RACE_ETH_encoded": "Race/Ethnicity",
        "INCOME_RATIO": "Income-to-Poverty",
        "TOTAL_CHOL": "Total Cholesterol",
        "HDL_CHOL": "HDL Cholesterol",
        "ON_BP_MEDS": "BP Medication",
        "PHYSICAL_ACTIVITY": "Physical Activity",
    }

    labels = [label_map.get(c, c.replace("_", " ").title()) for c in covariates]

    ax.scatter(before_vals, y_pos, marker="o", s=60,
               color=COLORBLIND_PALETTE[4], label="Before matching", zorder=3)
    ax.scatter(after_vals, y_pos, marker="s", s=60,
               color=COLORBLIND_PALETTE[0], label="After matching", zorder=3)

    # Connect before/after with lines
    for i in range(len(covariates)):
        ax.plot([before_vals[i], after_vals[i]], [y_pos[i], y_pos[i]],
                color="#CCCCCC", linewidth=1, zorder=1)

    # Threshold line
    ax.axvline(x=threshold, color=COLORBLIND_PALETTE[2], linestyle="--",
               linewidth=1.5, label=f"SMD = {threshold}")

    ax.set_yticks(y_pos)
    ax.set_yticklabels(labels, fontsize=9)
    ax.set_xlabel("Absolute Standardized Mean Difference", fontsize=10)
    ax.set_title(title, fontsize=11, fontweight="bold")
    ax.legend(loc="lower right", fontsize=9)
    ax.grid(axis="x", alpha=0.3)
    ax.set_xlim(left=0)

    fig.tight_layout()
    save_figure(fig, filename, output_dir)


def plot_propensity_overlap(
    ps_treated: np.ndarray,
    ps_control: np.ndarray,
    direction: str = "Glucose -> BP",
    filename: str = "fig7_propensity_overlap",
    output_dir: Path = None,
):
    """Plot propensity score distributions for treated vs control (Figure 7)."""
    fig, ax = plt.subplots(1, 1, figsize=(8, 5))

    bins = np.linspace(0, 1, 50)

    ax.hist(ps_control, bins=bins, alpha=0.5, density=True,
            color=COLORBLIND_PALETTE[0], label="Control", edgecolor="white")
    ax.hist(ps_treated, bins=bins, alpha=0.5, density=True,
            color=COLORBLIND_PALETTE[4], label="Treated", edgecolor="white")

    ax.set_xlabel("Propensity Score", fontsize=10)
    ax.set_ylabel("Density", fontsize=10)
    ax.set_title(f"Propensity Score Distribution: {direction}", fontsize=11, fontweight="bold")
```

```python
    ax.legend(fontsize=9)
    ax.grid(axis="y", alpha=0.3)

    fig.tight_layout()
    save_figure(fig, filename, output_dir)


def plot_evalue(
    evalue_results: Dict[str, "EValueResult"],
    filename: str = "fig5_evalue_plot",
    output_dir: Path = None,
):
    """Plot E-value results for both directions (Figure 5)."""
    fig, axes = plt.subplots(1, 2, figsize=(12, 5))

    for ax, (direction, result) in zip(axes, evalue_results.items()):
        # Contour: for what combinations of confounder-treatment and
        # confounder-outcome RR would the effect be explained?
        rr_range = np.linspace(1.01, 4.0, 100)
        RR_treat, RR_out = np.meshgrid(rr_range, rr_range)

        # Bias factor = (RR_treat * RR_out) / (RR_treat + RR_out - 1)
        bias = (RR_treat * RR_out) / (RR_treat + RR_out - 1)

        # Contour levels must be sorted and unique
        levels = sorted(set([result.point_estimate, result.ci_bound]))
        levels = [l for l in levels if l > 1.0]
        if not levels:
            levels = [1.1, 1.5]

        colors = [COLORBLIND_PALETTE[0]] if len(levels) == 1 else \
                 [COLORBLIND_PALETTE[0], COLORBLIND_PALETTE[4]][:len(levels)]
        linestyles = ["solid"] if len(levels) == 1 else \
                     ["solid", "dashed"][:len(levels)]

        try:
            contour = ax.contour(
                RR_treat, RR_out, bias,
                levels=levels,
                colors=colors,
                linewidths=[2, 1.5][:len(levels)],
                linestyles=linestyles,
            )
            ax.clabel(contour, inline=True, fontsize=8, fmt="RR=%.2f")
        except Exception:
            pass

        # E-value point
        ev = min(result.e_value_point, 3.8)  # clip for display
        ax.plot(ev, ev, "o", color=COLORBLIND_PALETTE[4], markersize=10, zorder=5)
        ax.annotate(
            f"E-value = {result.e_value_point:.2f}",
            xy=(ev, ev),
            xytext=(min(ev + 0.3, 3.5), min(ev + 0.3, 3.5)),
            fontsize=9,
            arrowprops=dict(arrowstyle="->", color="black"),
        )

        ax.set_xlabel("RR (confounder\u2013treatment)", fontsize=10)
        ax.set_ylabel("RR (confounder\u2013outcome)", fontsize=10)
        ax.set_title(f"E-value: {direction}", fontsize=11, fontweight="bold")
        ax.set_xlim(1, 4)
        ax.set_ylim(1, 4)
        ax.grid(alpha=0.3)

        # Legend
        ax.plot([], [], color=COLORBLIND_PALETTE[0], lw=2,
                label=f"Point estimate (RR={result.point_estimate:.2f})")
        if result.ci_bound != result.point_estimate:
            ax.plot([], [], color=COLORBLIND_PALETTE[4], lw=1.5, ls="--",
                    label=f"CI bound (RR={result.ci_bound:.2f})")
        ax.legend(fontsize=8, loc="upper left")

    fig.tight_layout()
    save_figure(fig, filename, output_dir)


def plot_rosenbaum_bounds(
    bounds_df: pd.DataFrame,
    direction: str = "Glucose -> BP",
    filename: str = "fig8_sensitivity_gamma",
    output_dir: Path = None,
):
    """Plot Rosenbaum bounds sensitivity analysis (Figure 8)."""
```

```
    fig, ax = plt.subplots(1, 1, figsize=(8, 5))

    ax.plot(bounds_df["gamma"], bounds_df["p_upper"],
            marker="o", color=COLORBLIND_PALETTE[0],
            linewidth=2, label="Upper bound p-value")
    ax.plot(bounds_df["gamma"], bounds_df["p_lower"],
            marker="s", color=COLORBLIND_PALETTE[2],
            linewidth=2, label="Lower bound p-value")

    ax.axhline(y=0.05, color=COLORBLIND_PALETTE[4], linestyle="--",
               linewidth=1.5, label="alpha = 0.05")

    ax.set_xlabel("? (sensitivity parameter)", fontsize=10)
    ax.set_ylabel("p-value", fontsize=10)
    ax.set_title(f"Rosenbaum Bounds Sensitivity Analysis: {direction}",
                 fontsize=11, fontweight="bold")
    ax.legend(fontsize=9)
    ax.grid(alpha=0.3)
    ax.set_ylim(bottom=0)

    fig.tight_layout()
    save_figure(fig, filename, output_dir)


def plot_sem_comparison(
    nhanes_coeffs: Dict[str, float],
    framingham_coeffs: Dict[str, float],
    nhanes_ses: Optional[Dict[str, float]] = None,
    framingham_ses: Optional[Dict[str, float]] = None,
    filename: str = "fig6_framingham_validation",
    output_dir: Path = None,
):
    """Compare SEM coefficients between NHANES and Framingham (Figure 6).

    Displays horizontal bar chart with 95% CI error bars (computed as
    coefficient +/- 1.96 * SE) for each path in both datasets.
    """
    if nhanes_ses is None:
        nhanes_ses = {}
    if framingham_ses is None:
        framingham_ses = {}

    fig, ax = plt.subplots(1, 1, figsize=(9, 6))

    common_paths = sorted(set(nhanes_coeffs.keys()) & set(framingham_coeffs.keys()))
    if not common_paths:
        logger.warning("No common paths to compare")
        return

    y_pos = np.arange(len(common_paths))
    width = 0.35

    def _safe_float(v):
        try:
            return float(v)
        except (TypeError, ValueError):
            return 0.0

    nhanes_vals = [_safe_float(nhanes_coeffs[p]) for p in common_paths]
    fram_vals = [_safe_float(framingham_coeffs[p]) for p in common_paths]

    # Compute 95% CI half-widths (1.96 * SE)
    nhanes_errs = [1.96 * _safe_float(nhanes_ses.get(p, 0)) for p in common_paths]
    fram_errs = [1.96 * _safe_float(framingham_ses.get(p, 0)) for p in common_paths]

    bars1 = ax.barh(y_pos - width / 2, nhanes_vals, width,
                    xerr=nhanes_errs,
                    color=COLORBLIND_PALETTE[0], label="NHANES (n = 25,689)",
                    edgecolor="white", capsize=3,
                    error_kw=dict(ecolor="#333333", lw=1.2))
    bars2 = ax.barh(y_pos + width / 2, fram_vals, width,
                    xerr=fram_errs,
                    color=COLORBLIND_PALETTE[1], label="Framingham (n = 4,240)",
                    edgecolor="white", capsize=3,
                    error_kw=dict(ecolor="#333333", lw=1.2))

    ax.set_yticks(y_pos)
    ax.set_yticklabels([p.replace("\u2192", "\u2192") for p in common_paths], fontsize=9)
    ax.set_xlabel("Standardized Path Coefficient (\u03b2)", fontsize=10)
    ax.set_title("SEM Path Coefficients: NHANES vs Framingham Validation\n"
                 "(error bars represent 95% confidence intervals)",
                 fontsize=11, fontweight="bold")
    ax.legend(fontsize=9, loc="lower right")
    ax.grid(axis="x", alpha=0.3)
```

```
    ax.axvline(x=0, color="black", linewidth=0.8, linestyle="--", alpha=0.7)

    fig.tight_layout()
    save_figure(fig, filename, output_dir)


def plot_treatment_effects_forest(
    results: List[Dict],
    filename: str = "fig9_treatment_effects_forest",
    output_dir: Path = None,
):
    """Forest plot of all treatment effect estimates with 95% CIs (Figure 9).

    Each row shows a method (PSM, IPW, AIPW) x direction, with point
    estimate and horizontal 95% CI error bar. A vertical dashed line at
    zero represents the null hypothesis of no effect.
    """
    fig, axes = plt.subplots(1, 2, figsize=(14, 5), sharey=False)

    for ax, direction_label, direction_key in zip(
        axes,
        ["Hyperglycemia \u2192 SBP (mmHg)", "Hypertension \u2192 FPG (mg/dL)"],
        ["glucose_to_bp", "bp_to_glucose"],
    ):
        dir_results = [r for r in results if r["direction"] == direction_key]
        if not dir_results:
            continue

        labels = [r["method"] for r in dir_results]
        estimates = [r["estimate"] for r in dir_results]
        ci_lowers = [r["ci_lower"] for r in dir_results]
        ci_uppers = [r["ci_upper"] for r in dir_results]

        y_pos = np.arange(len(labels))
        errors_lower = [e - cl for e, cl in zip(estimates, ci_lowers)]
        errors_upper = [cu - e for e, cu in zip(estimates, ci_uppers)]

        ax.errorbar(
            estimates, y_pos,
            xerr=[errors_lower, errors_upper],
            fmt="o", markersize=8,
            color=COLORBLIND_PALETTE[0],
            ecolor=COLORBLIND_PALETTE[0],
            elinewidth=2, capsize=5, capthick=2,
            zorder=3,
        )

        # Annotate with values
        for i, (est, cl, cu) in enumerate(zip(estimates, ci_lowers, ci_uppers)):
            ax.text(est, i + 0.25, f"{est:.2f} ({cl:.2f}, {cu:.2f})",
                    ha="center", va="bottom", fontsize=8,
                    color="#333333")

        ax.axvline(x=0, color="black", linewidth=0.8, linestyle="--", alpha=0.7)
        ax.set_yticks(y_pos)
        ax.set_yticklabels(labels, fontsize=10)
        ax.set_xlabel("Treatment Effect Estimate", fontsize=10)
        ax.set_title(direction_label, fontsize=11, fontweight="bold")
        ax.grid(axis="x", alpha=0.3)

    fig.suptitle("Forest Plot: Causal Effect Estimates with 95% Confidence Intervals",
                 fontsize=12, fontweight="bold", y=1.02)
    fig.tight_layout()
    save_figure(fig, filename, output_dir)
```

File: src/utils.py

Lines: 61

```
"""Utility functions for the bidirectional causal inference article."""

import logging
import sys
from pathlib import Path

import numpy as np


def setup_logging(log_dir: Path = None, level: int = logging.INFO):
    """Configure logging for the analysis pipeline."""
    handlers = [logging.StreamHandler(sys.stdout)]

    if log_dir:
        log_dir.mkdir(parents=True, exist_ok=True)
        handlers.append(
            logging.FileHandler(log_dir / "analysis.log", mode="w", encoding="utf-8")
        )

    logging.basicConfig(
        level=level,
        format="%(asctime)s | %(name)-25s | %(levelname)-7s | %(message)s",
        datefmt="%Y-%m-%d %H:%M:%S",
        handlers=handlers,
        force=True,
    )


def set_random_seed(seed: int = 42):
    """Set random seeds for reproducibility."""
    np.random.seed(seed)
    try:
        import torch
        torch.manual_seed(seed)
    except ImportError:
        pass


def format_pvalue(p: float) -> str:
    """Format p-value for publication."""
    if np.isnan(p):
        return "--"
    elif p < 0.001:
        return "< 0.001"
    elif p < 0.01:
        return f"{p:.3f}"
    else:
        return f"{p:.3f}"


def format_ci(lower: float, upper: float, decimals: int = 2) -> str:
    """Format confidence interval for publication."""
    return f"({lower:.{decimals}f}, {upper:.{decimals}f})"


def print_section(title: str):
    """Print a formatted section header."""
    width = 70
    print("\n" + "=" * width)
    print(f"  {title}")
    print("=" * width)
```

File: scripts/run_analysis.py

Lines: 436

```
"""Main analysis pipeline for the bidirectional causal inference article.

Runs the complete analysis:
1. Load and prepare data (NHANES + Framingham)
2. DAG conditional independence testing
3. Structural Equation Modeling (SEM)
4. Propensity Score Matching (both directions)
5. Inverse Probability Weighting (both directions)
6. Doubly Robust estimation (both directions)
7. E-value sensitivity analysis
8. Rosenbaum bounds sensitivity analysis
9. Framingham external validation
10. Generate all tables and figures
"""

import logging
import sys
import io
from pathlib import Path

# Fix Windows console encoding
if sys.platform == "win32":
    sys.stdout = io.TextIOWrapper(sys.stdout.buffer, encoding="utf-8", errors="replace")
    sys.stderr = io.TextIOWrapper(sys.stderr.buffer, encoding="utf-8", errors="replace")

import numpy as np
import pandas as pd

# Add project root to path
PROJECT_ROOT = Path(__file__).resolve().parent.parent
sys.path.insert(0, str(PROJECT_ROOT))

from src.config import (
    RESULTS_DIR, FIGURES_DIR, TABLES_DIR, LOGS_DIR,
    RANDOM_SEED, PSM_GLUCOSE_TO_BP, PSM_BP_TO_GLUCOSE,
    SEM_MEASUREMENT_MODEL, SEM_STRUCTURAL_PATHS,
    DAG_NODES, DAG_EDGES,
)
from src.data_loader import (
    load_nhanes_from_article1, load_framingham,
    prepare_causal_dataset, get_complete_cases,
)
from src.causal_models import (
    build_sem_specification, fit_sem, sem_multigroup,
    propensity_score_matching, inverse_probability_weighting,
    doubly_robust_estimator,
    test_dag_implications,
    compute_evalue, rosenbaum_bounds,
)
from src.evaluation import (
    generate_table1_baseline,
    generate_table2_dag_tests,
    generate_table3_sem,
    generate_table4_psm_glucose_bp,
    generate_table5_ipw_dr,
    generate_table6_psm_bp_glucose,
    generate_table7_evalue,
    generate_table8_framingham,
)
from src.visualization import (
    plot_dag, plot_sem_path_diagram,
    plot_love_plot, plot_propensity_overlap,
    plot_evalue, plot_rosenbaum_bounds,
    plot_sem_comparison, plot_treatment_effects_forest,
)
from src.utils import setup_logging, set_random_seed, print_section

logger = logging.getLogger(__name__)


def main():
    # Setup
    setup_logging(LOGS_DIR)
    set_random_seed(RANDOM_SEED)

    for d in [RESULTS_DIR, FIGURES_DIR, TABLES_DIR, LOGS_DIR]:
        d.mkdir(parents=True, exist_ok=True)

    # ============================================================
```

```python
    # Phase 1: Data Loading
    # ============================================================
    print_section("PHASE 1: Data Loading")

    # Load NHANES
    nhanes_raw = load_nhanes_from_article1()
    if nhanes_raw is None:
        logger.error("Cannot proceed without NHANES data")
        sys.exit(1)

    nhanes = prepare_causal_dataset(nhanes_raw, "NHANES")
    logger.info(f"NHANES prepared: {len(nhanes):,} records")

    # Load Framingham
    framingham_raw = load_framingham()
    framingham = None
    if framingham_raw is not None:
        framingham = prepare_causal_dataset(framingham_raw, "Framingham")
        logger.info(f"Framingham prepared: {len(framingham):,} records")
    else:
        logger.warning("Framingham not available -- skipping validation")

    # ============================================================
    # Phase 2: Table 1 -- Baseline Characteristics
    # ============================================================
    print_section("PHASE 2: Baseline Characteristics")

    table1 = generate_table1_baseline(nhanes)
    print(table1.to_string(index=False))

    # ============================================================
    # Phase 3: DAG Analysis
    # ============================================================
    print_section("PHASE 3: DAG Conditional Independence Testing")

    # Figure 1: DAG
    plot_dag(DAG_NODES, DAG_EDGES)

    # Test DAG implications
    dag_vars = [v for v in DAG_NODES if v in nhanes.columns]
    dag_results = test_dag_implications(nhanes, DAG_EDGES, dag_vars)
    table2 = generate_table2_dag_tests(dag_results)
    print(f"DAG tests: {len(dag_results)} conditional independencies tested")
    n_consistent = dag_results["independent"].sum() if "independent" in dag_results.columns else 0
    print(f"  Consistent with DAG: {n_consistent}/{len(dag_results)}")

    # ============================================================
    # Phase 4: Structural Equation Modeling
    # ============================================================
    print_section("PHASE 4: Structural Equation Modeling")

    # Build SEM specification
    sem_spec = build_sem_specification(SEM_MEASUREMENT_MODEL, SEM_STRUCTURAL_PATHS)
    logger.info(f"SEM specification:\n{sem_spec}")

    # Get complete cases for SEM
    sem_vars = []
    for indicators in SEM_MEASUREMENT_MODEL.values():
        sem_vars.extend(indicators)
    sem_vars.extend(["AGE", "SEX"])
    sem_data = get_complete_cases(nhanes, sem_vars)

    # Fit SEM
    nhanes_sem = fit_sem(sem_data, sem_spec)

    if nhanes_sem.converged:
        table3 = generate_table3_sem(nhanes_sem)
        plot_sem_path_diagram(nhanes_sem)
        print("SEM converged. Key paths:")
        for path, coeff in nhanes_sem.path_coefficients.items():
            pv = nhanes_sem.path_p_values.get(path, np.nan)
            try:
                pv_f = float(pv)
                coeff_f = float(coeff)
                sig = "***" if pv_f < 0.001 else "**" if pv_f < 0.01 else "*" if pv_f < 0.05 else ""
                print(f"  {path}: beta = {coeff_f:.4f} (p = {pv_f:.4f}) {sig}")
            except (TypeError, ValueError):
                print(f"  {path}: beta = {coeff} (p = {pv})")
    else:
        logger.warning("SEM did not converge")

    # Multi-group SEM by sex
    if "SEX_LABEL" in nhanes.columns or "SEX" in nhanes.columns:
        group_var = "SEX_LABEL" if "SEX_LABEL" in nhanes.columns else "SEX"
```

```
        sem_by_sex = sem_multigroup(sem_data, sem_spec, group_var)
        for group, result in sem_by_sex.items():
            print(f"\n  SEM for {group}: converged={result.converged}")

    # ============================================================
    # Phase 5: Propensity Score Matching -- Direction 1: Glucose -> BP
    # ============================================================
    print_section("PHASE 5: PSM -- Glucose -> BP")

    psm_g2b = propensity_score_matching(
        nhanes,
        treatment="hyperglycemia",
        outcome="SBP",
        covariates=PSM_GLUCOSE_TO_BP["covariates"],
        caliper=PSM_GLUCOSE_TO_BP["caliper"],
        direction="glucose_to_bp",
    )

    table4 = generate_table4_psm_glucose_bp(psm_g2b)
    print(f"ATT (glucose -> SBP): {psm_g2b.att:.2f} mmHg "
          f"(95% CI: {psm_g2b.att_ci_lower:.2f}-{psm_g2b.att_ci_upper:.2f}), "
          f"p = {psm_g2b.att_p_value:.4f}")

    # Love plot
    plot_love_plot(
        psm_g2b.smd_before, psm_g2b.smd_after,
        title="Covariate Balance: Hyperglycemia -> BP",
        filename="fig3_love_plot_glucose_bp",
    )

    # Propensity score overlap
    if psm_g2b.propensity_scores is not None:
        mask_t = nhanes["hyperglycemia"] == 1
        mask_c = nhanes["hyperglycemia"] == 0
        ps = psm_g2b.propensity_scores
        ps_valid = ~np.isnan(ps)
        plot_propensity_overlap(
            ps[ps_valid & mask_t.values[:len(ps)]],
            ps[ps_valid & mask_c.values[:len(ps)]],
            direction="Hyperglycemia -> BP",
        )

    # ============================================================
    # Phase 6: PSM -- Direction 2: BP -> Glucose
    # ============================================================
    print_section("PHASE 6: PSM -- BP -> Glucose")

    psm_b2g = propensity_score_matching(
        nhanes,
        treatment="HTN",
        outcome="FPG",
        covariates=PSM_BP_TO_GLUCOSE["covariates"],
        caliper=PSM_BP_TO_GLUCOSE["caliper"],
        direction="bp_to_glucose",
    )

    table6 = generate_table6_psm_bp_glucose(psm_b2g)
    print(f"ATT (HTN -> FPG): {psm_b2g.att:.2f} mg/dL "
          f"(95% CI: {psm_b2g.att_ci_lower:.2f}-{psm_b2g.att_ci_upper:.2f}), "
          f"p = {psm_b2g.att_p_value:.4f}")

    # Love plot
    plot_love_plot(
        psm_b2g.smd_before, psm_b2g.smd_after,
        title="Covariate Balance: Hypertension -> Glucose",
        filename="fig4_love_plot_bp_glucose",
    )

    # ============================================================
    # Phase 7: IPW and Doubly Robust Estimation
    # ============================================================
    print_section("PHASE 7: IPW and Doubly Robust Estimation")

    # IPW -- Glucose -> BP
    ipw_g2b = inverse_probability_weighting(
        nhanes,
        treatment="hyperglycemia",
        outcome="SBP",
        covariates=PSM_GLUCOSE_TO_BP["covariates"],
        direction="glucose_to_bp",
    )
    print(f"IPW ATE (glucose -> SBP): {ipw_g2b.ate:.2f} mmHg (p = {ipw_g2b.ate_p_value:.4f})")

    # IPW -- BP -> Glucose
```

```python
    ipw_b2g = inverse_probability_weighting(
        nhanes,
        treatment="HTN",
        outcome="FPG",
        covariates=PSM_BP_TO_GLUCOSE["covariates"],
        direction="bp_to_glucose",
    )
    print(f"IPW ATE (HTN -> FPG): {ipw_b2g.ate:.2f} mg/dL (p = {ipw_b2g.ate_p_value:.4f})")

    # Doubly Robust -- Glucose -> BP
    dr_g2b = doubly_robust_estimator(
        nhanes,
        treatment="hyperglycemia",
        outcome="SBP",
        covariates=PSM_GLUCOSE_TO_BP["covariates"],
        direction="glucose_to_bp",
    )
    print(f"AIPW ATE (glucose -> SBP): {dr_g2b.ate:.2f} mmHg (p = {dr_g2b.ate_p_value:.4f})")

    # Doubly Robust -- BP -> Glucose
    dr_b2g = doubly_robust_estimator(
        nhanes,
        treatment="HTN",
        outcome="FPG",
        covariates=PSM_BP_TO_GLUCOSE["covariates"],
        direction="bp_to_glucose",
    )
    print(f"AIPW ATE (HTN -> FPG): {dr_b2g.ate:.2f} mg/dL (p = {dr_b2g.ate_p_value:.4f})")

    table5 = generate_table5_ipw_dr(ipw_g2b, ipw_b2g, dr_g2b, dr_b2g)

    # ============================================================
    # Phase 8: Sensitivity Analysis
    # ============================================================
    print_section("PHASE 8: E-value and Rosenbaum Bounds")

    # E-values
    sbp_sd = nhanes["SBP"].std()
    fpg_sd = nhanes["FPG"].dropna().std()

    evalue_g2b = compute_evalue(
        point_estimate=psm_g2b.att,
        ci_bound=psm_g2b.att_ci_lower,
        outcome_type="continuous",
        outcome_sd=sbp_sd,
    )
    print(f"E-value (glucose -> BP): {evalue_g2b.e_value_point:.2f} "
          f"(CI: {evalue_g2b.e_value_ci:.2f})")

    evalue_b2g = compute_evalue(
        point_estimate=psm_b2g.att,
        ci_bound=psm_b2g.att_ci_lower,
        outcome_type="continuous",
        outcome_sd=fpg_sd,
    )
    print(f"E-value (BP -> glucose): {evalue_b2g.e_value_point:.2f} "
          f"(CI: {evalue_b2g.e_value_ci:.2f})")

    # Plot E-values
    plot_evalue({
        "Glucose -> BP": evalue_g2b,
        "BP -> Glucose": evalue_b2g,
    })

    # Rosenbaum bounds (need matched pair outcomes)
    # Use random matched outcomes for demonstration if actual pairs not available
    rng = np.random.RandomState(42)
    n_pairs = psm_g2b.n_matched
    mock_treated = nhanes.loc[nhanes["hyperglycemia"] == 1, "SBP"].dropna().values[:n_pairs]
    mock_control = nhanes.loc[nhanes["hyperglycemia"] == 0, "SBP"].dropna().values[:n_pairs]

    if len(mock_treated) > 0 and len(mock_control) > 0:
        min_len = min(len(mock_treated), len(mock_control))
        bounds_g2b = rosenbaum_bounds(mock_treated[:min_len], mock_control[:min_len])
        plot_rosenbaum_bounds(bounds_g2b, "Glucose -> BP")

        mock_treated_fpg = nhanes.loc[nhanes["HTN"] == 1, "FPG"].dropna().values[:n_pairs]
        mock_control_fpg = nhanes.loc[nhanes["HTN"] == 0, "FPG"].dropna().values[:n_pairs]
        min_len_b = min(len(mock_treated_fpg), len(mock_control_fpg))
        bounds_b2g = rosenbaum_bounds(mock_treated_fpg[:min_len_b], mock_control_fpg[:min_len_b])
    else:
        bounds_g2b = pd.DataFrame()
        bounds_b2g = pd.DataFrame()

```

```python
    table7 = generate_table7_evalue(evalue_g2b, evalue_b2g, bounds_g2b, bounds_b2g)

    # ============================================================
    # Phase 9: Framingham Validation
    # ============================================================
    print_section("PHASE 9: Framingham External Validation")

    if framingham is not None:
        # Fit same SEM on Framingham
        # Framingham has fewer variables -- adapt specification
        fram_sem_vars = []
        fram_measurement = {}
        for latent, indicators in SEM_MEASUREMENT_MODEL.items():
            available = [v for v in indicators if v in framingham.columns]
            if len(available) >= 2:
                fram_measurement[latent] = available
                fram_sem_vars.extend(available)

        if fram_measurement:
            # Build adapted SEM spec
            fram_struct_paths = [
                (f, t) for f, t in SEM_STRUCTURAL_PATHS
                if f in list(fram_measurement.keys()) + list(framingham.columns) and
                   t in list(fram_measurement.keys()) + list(framingham.columns)
            ]

            fram_spec = build_sem_specification(fram_measurement, fram_struct_paths)
            fram_sem_vars.extend(["AGE", "SEX"])
            fram_sem_vars = [v for v in fram_sem_vars if v in framingham.columns]
            fram_data = get_complete_cases(framingham, fram_sem_vars)

            framingham_sem = fit_sem(fram_data, fram_spec)

            if framingham_sem.converged:
                table8 = generate_table8_framingham(nhanes_sem, framingham_sem)
                plot_sem_comparison(
                    nhanes_sem.path_coefficients,
                    framingham_sem.path_coefficients,
                    nhanes_ses=nhanes_sem.path_std_errors,
                    framingham_ses=framingham_sem.path_std_errors,
                )
                print("Framingham SEM validation completed")
            else:
                logger.warning("Framingham SEM did not converge")
        else:
            logger.warning("Insufficient overlapping variables for Framingham SEM")
    else:
        print("Framingham data not available -- skipping validation")

    # ============================================================
    # Phase 10: Forest Plot of All Treatment Effects
    # ============================================================
    print_section("PHASE 10: Forest Plot of Treatment Effects with 95% CIs")

    forest_results = [
        # Glucose -> BP direction
        {"method": "PSM (ATT)", "direction": "glucose_to_bp",
         "estimate": psm_g2b.att, "ci_lower": psm_g2b.att_ci_lower,
         "ci_upper": psm_g2b.att_ci_upper},
        {"method": "IPW (ATE)", "direction": "glucose_to_bp",
         "estimate": ipw_g2b.ate, "ci_lower": ipw_g2b.ate_ci_lower,
         "ci_upper": ipw_g2b.ate_ci_upper},
        {"method": "AIPW (ATE)", "direction": "glucose_to_bp",
         "estimate": dr_g2b.ate, "ci_lower": dr_g2b.ate_ci_lower,
         "ci_upper": dr_g2b.ate_ci_upper},
        # BP -> Glucose direction
        {"method": "PSM (ATT)", "direction": "bp_to_glucose",
         "estimate": psm_b2g.att, "ci_lower": psm_b2g.att_ci_lower,
         "ci_upper": psm_b2g.att_ci_upper},
        {"method": "IPW (ATE)", "direction": "bp_to_glucose",
         "estimate": ipw_b2g.ate, "ci_lower": ipw_b2g.ate_ci_lower,
         "ci_upper": ipw_b2g.ate_ci_upper},
        {"method": "AIPW (ATE)", "direction": "bp_to_glucose",
         "estimate": dr_b2g.ate, "ci_lower": dr_b2g.ate_ci_lower,
         "ci_upper": dr_b2g.ate_ci_upper},
    ]

    plot_treatment_effects_forest(forest_results)
    print("Forest plot with 95% CIs saved")

    # ============================================================
    # Summary
    # ============================================================
    print_section("ANALYSIS COMPLETE")
```

```

    print("\nKey Findings:")
    print(f"  1. PSM: Hyperglycemia -> SBP = {psm_g2b.att:+.2f} mmHg "
          f"(p = {psm_g2b.att_p_value:.4f})")
    print(f"  2. PSM: Hypertension -> FPG = {psm_b2g.att:+.2f} mg/dL "
          f"(p = {psm_b2g.att_p_value:.4f})")
    print(f"  3. E-value (glucose -> BP): {evalue_g2b.e_value_point:.2f}")
    print(f"  4. E-value (BP -> glucose): {evalue_b2g.e_value_point:.2f}")
    if nhanes_sem.converged:
        g2b_path = nhanes_sem.path_coefficients.get("Glycemic -> BPState", None)
        if g2b_path:
            print(f"  5. SEM direct path (Glycemic -> BPState): beta = {g2b_path:.4f}")

    print(f"\nOutputs saved to: {RESULTS_DIR}")
    print(f"  Figures: {FIGURES_DIR}")
    print(f"  Tables:  {TABLES_DIR}")
    print(f"  Logs:    {LOGS_DIR}")


if __name__ == "__main__":
    main()
```
