# Supplementary material for: Bidirectional Associations Between Blood Glucose and Blood Pressure: A Data-Driven Causal Analysis Using Structural Equation Modelling and Granger Causality on NHANES Longitudinal Data
Source: J Clin Med. 2026 May 13;15(10):3751. doi: 10.3390/jcm15103751 (PMC13207677; doi:10.3390/jcm15103751)
Supplement: Supplementary file 1 [file jcm-15-03751-s001.zip › Supplement_S2_Extended_DAG_Tests.pdf]

Supplementary Material S2: Extended DAG Conditional Independence Tests

Bidirectional Associations Between Blood Glucose and Blood Pressure: A Data-Driven Causal Analysis Using Structural Equation Modeling and Granger Causality on NHANES Longitudinal Data

The main article (Table 2) reports a selected subset of the 51 conditional independencies implied by the a priori DAG. This supplement provides the complete list of all 51 tests together with the conditioning set, partial correlation coefficient, p-value, and a decision flag indicating whether the observed dependence is consistent with the DAG at a Bonferroni-corrected threshold (p > 0.05). Five tests are consistent with the DAG structure; 46 are not. See the main article Section 3.2 for discussion.

Variable X	Variable Y	Conditioning Set	Partial r	p-value	Independent (p > 0.05)
FPG	HBA1C	BMI, SEX, HOMA_IR, AGE, CRP	0.800	0.0000	False
FPG	WAIST	BMI, SEX, HOMA_IR, AGE, CRP	0.043	0.0000	False
FPG	ON_BP_MEDS	BMI, SEX, HOMA_IR, AGE, CRP	0.078	0.0000	False
FPG	TOTAL_CHOL	BMI, SEX, HOMA_IR, AGE, CRP	0.021	0.0034	False
FPG	HDL_CHOL	BMI, SEX, HOMA_IR, AGE, CRP	−0.084	0.0000	False
HBA1C	WAIST	AGE	0.202	0.0000	False
HBA1C	HOMA_IR	AGE, BMI, WAIST	0.289	0.0000	False
HBA1C	SEX	AGE	−0.034	0.0000	False
HBA1C	ON_BP_MEDS	AGE	0.118	0.0000	False
HBA1C	TOTAL_CHOL	AGE	0.019	0.0000	False
HBA1C	HDL_CHOL	AGE	−0.174	0.0000	False
SBP	WAIST	HDL_CHOL, TOTAL_CHOL, BMI, HBA1C, SEX, HOMA_IR, AGE, ON_BP_MEDS, CRP, FPG	−0.041	0.0000	False
DBP	HBA1C	BMI, SEX, AGE, ON_BP_MEDS, FPG	−0.010	0.1180	True
DBP	SBP	HDL_CHOL, TOTAL_CHOL, BMI, HBA1C, SEX, HOMA_IR, ON_BP_MEDS, AGE, CRP, FPG	0.384	0.0000	False
DBP	WAIST	BMI, SEX, AGE, ON_BP_MEDS, FPG	0.001	0.9100	True
DBP	HOMA_IR	BMI, SEX, AGE, ON_BP_MEDS, WAIST, FPG	−0.022	0.0004	False
DBP	TOTAL_CHOL	BMI, SEX, AGE, ON_BP_MEDS, FPG	0.141	0.0000	False
DBP	HDL_CHOL	BMI, SEX, AGE, ON_BP_MEDS, FPG	0.017	0.0061	False
BMI	HBA1C	WAIST, AGE, SEX	0.020	0.0000	False
BMI	ON_BP_MEDS	WAIST, AGE, SEX	0.033	0.0000	False
BMI	TOTAL_CHOL	WAIST, AGE, SEX	−0.039	0.0000	False
BMI	HDL_CHOL	WAIST, AGE, SEX	−0.008	0.0848	True
BMI	CRP	WAIST, AGE, SEX	0.052	0.0000	False
HOMA_IR	SEX	BMI, WAIST, AGE	−0.007	0.2518	True
HOMA_IR	ON_BP_MEDS	BMI, WAIST, AGE	0.052	0.0000	False
HOMA_IR	TOTAL_CHOL	BMI, WAIST, AGE	−0.038	0.0000	False

Variable X	Variable Y	Conditioning Set	Partial r	p-value	Independent (p > 0.05)
AGE	WAIST	(none)	—	0.0000	False
AGE	SEX	(none)	—	0.0002	False
AGE	ON_BP_MEDS	(none)	—	0.0000	False
AGE	TOTAL_CHOL	(none)	—	0.0000	False
AGE	HDL_CHOL	(none)	—	0.0000	False
AGE	CRP	(none)	—	0.0000	False
SEX	WAIST	(none)	—	0.0000	False
SEX	TOTAL_CHOL	(none)	—	0.0000	False
ON_BP_MEDS	WAIST	(none)	—	0.0000	False
ON_BP_MEDS	SEX	(none)	—	0.0004	False
ON_BP_MEDS	TOTAL_CHOL	(none)	—	0.1459	True
TOTAL_CHOL	WAIST	(none)	—	0.0000	False
HDL_CHOL	WAIST	(none)	—	0.0000	False
HDL_CHOL	HOMA_IR	BMI, WAIST, AGE	−0.102	0.0000	False
HDL_CHOL	SEX	(none)	—	0.0000	False
HDL_CHOL	ON_BP_MEDS	(none)	—	0.0000	False
HDL_CHOL	TOTAL_CHOL	(none)	—	0.0000	False
CRP	HBA1C	AGE	0.104	0.0000	False
CRP	DBP	BMI, SEX, AGE, ON_BP_MEDS, FPG	0.015	0.0420	False
CRP	WAIST	(none)	—	0.0000	False
CRP	HOMA_IR	BMI, WAIST, AGE	0.036	0.0000	False
CRP	SEX	(none)	—	0.0000	False
CRP	ON_BP_MEDS	(none)	—	0.0000	False
CRP	TOTAL_CHOL	(none)	—	0.0000	False
CRP	HDL_CHOL	(none)	—	0.0000	False

Note. Partial correlations computed controlling for the adjustment set specified by d-separation on the a priori DAG. p-values from Fisher's z-transformation of the partial correlation. A dash (—) in the Partial r column indicates that the conditioning set was empty (marginal test). Rows with Independent = True are the five conditional independencies consistent with the DAG: DBP–HBA1C, DBP–WAIST, BMI–HDL_CHOL, HOMA_IR–SEX, and ON_BP_MEDS–TOTAL_CHOL. All analyses used the complete-case SEM subsample (n = 25,689) with the corrected SMOKING covariate.
