# Supplementary material for: Bidirectional Associations Between Blood Glucose and Blood Pressure: A Data-Driven Causal Analysis Using Structural Equation Modelling and Granger Causality on NHANES Longitudinal Data
Source: J Clin Med. 2026 May 13;15(10):3751. doi: 10.3390/jcm15103751 (PMC13207677; doi:10.3390/jcm15103751)
Supplement: Supplementary file 1 [file jcm-15-03751-s001.zip › Supplement_S3_Workflow_Diagram.pdf]

Supplement S3. Analytical Pipeline Schematic

Bidirectional Associations Between Blood Glucose and Blood Pressure: A Data-Driven Causal Analysis Using SEM and Granger Causality on NHANES Longitudinal Data

This supplement provides a single-page schematic of the end-to-end analytical pipeline used in the study. It is intended to support readers who wish to follow the sequence of decisions and the flow of evidence from raw NHANES data through the triangulated causal interpretation, complementing the prose in Sections 2.2-2.7 of the main text.

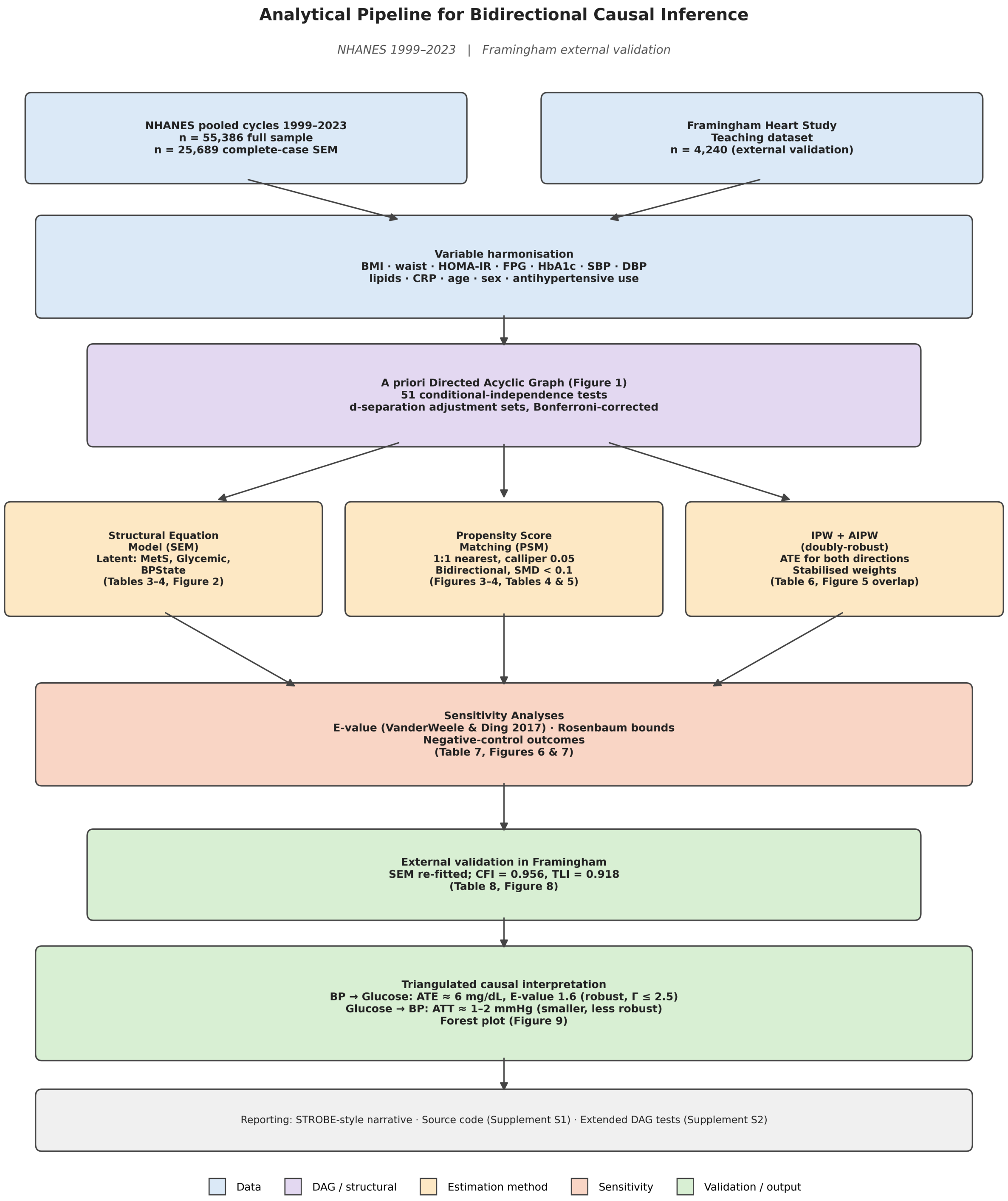

Figure S1. Analytical pipeline for bidirectional causal inference. Two harmonised data sources (NHANES 1999-2023 and the Framingham Heart Study teaching dataset) feed an a priori directed acyclic graph (DAG)

whose 51 implied conditional independencies are tested via partial correlation with d-separated adjustment sets. Three estimation strands are then run in parallel on the harmonised covariate set: a structural equation model with three latent constructs (MetS, Glycemic, BPState), 1:1 propensity-score matching in both causal directions, and inverse-probability weighting with its doubly-robust AIPW extension. Each estimator is followed by sensitivity diagnostics (E-values, Rosenbaum bounds and negative-control outcomes); the structural model is then re-fitted in Framingham as an external replication; and the converging estimates are summarised in a triangulated causal interpretation reported alongside open source code (Supplement S1) and extended DAG diagnostics (Supplement S2).

Notes on reading the schematic

Coloured swim-lanes group functionally related steps: blue = data preparation, purple = structural / DAG specification, orange = estimation, salmon = sensitivity analyses, green = validation and reporting outputs. Arrows denote the order of operations rather than information flow within statistical models. Sample sizes shown in the data boxes refer to the analytical samples after the inclusion criteria described in Section 2.1.
